# Perturbed synaptic adhesion molecules signaling in neurodevelopmental disorders: The impact of metal neurotoxicity

**DOI:** 10.1002/ibra.70005

**Published:** 2025-11-04

**Authors:** Toheeb O. Oyerinde, Victor E. Anadu, Tobiloba S. Olajide, Ileje I. Ukwubile, Olayemi K. Ijomone, Kingsley A. Iteire, Michael Aschner, Omamuyovwi M. Ijomone

**Affiliations:** ^1^ Laboratory for Experimental and Translational Neurobiology University of Medical Sciences Ondo Nigeria; ^2^ Department of Anatomy, Faculty of Basic Medical Sciences University of Medical Sciences Ondo Nigeria; ^3^ Department of Molecular Pharmacology Albert Einstein College of Medicine Bronx New York USA

**Keywords:** neurodevelopmental disorders, neurotoxic metals, synapses and autism, synaptic adhesion molecules

## Abstract

Synaptic adhesion molecules (SAMs) are glycoproteins localized on neuronal surfaces, primarily expressed at synaptic plasma membranes. SAMs play a role in inducing formation, maturation, plasticity, and assembly of synaptic connections, which are vital for normal neurodevelopment. SAMs link the pre‐ and post‐synaptic compartments and assist in inter‐synaptic signaling and recognition. An increasing variety of SAMs, including neurexins and neuroligins, immunoglobulin (Ig) domain proteins‐like synaptic cell adhesion molecule (SynCAM) and neural cell adhesion molecule, receptor phosphotyrosine kinases and phosphatases, as well as various leucine‐rich repeat proteins, have been identified. Neurodevelopmental disorders (NDDs), like autism, attention deficit hyperactivity disorder, intellectual disabilities, and cerebral palsy, have been associated with altered SAMs. NDDs are characterized by a spectrum of challenges stemming from abnormal brain development. The etiology of these disorders involves the interaction between genes and environmental factors, such as metals. This review aims to provide a comprehensive overview of the literature, highlighting the role of SAMs in NDDs and potential mechanisms via which neurotoxic metals may contribute to the pathogenesis of NDDs that could involve perturbations in SAMs. Understanding these interconnections will assist in identifying therapeutic targets for these disorders.

## Graphical abstract

Synaptic adhesion molecules (SAM) are essential for the formation, maturation, function, plasticity, and assembly of synaptic connections, all critical for normal neurodevelopment. Excessive exposure to neurotoxic metals during development could disrupt the developing brain and potentially lead to neurodevelopmental disorders (NDDs). Overexposure to these metals can also interfere with SAM signaling, and disruptions in these pathways are frequently linked to NDDs. In this article, we posit a possible interconnection between metal neurotoxicity and SAM perturbation in the etiopathogenesis of NDDs.

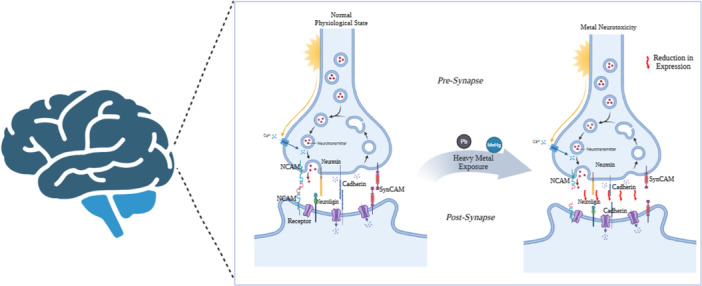

## 1 INTRODUCTION

Synapses, which are asymmetric junctions between neurons involving three key structures—the presynaptic and postsynaptic membranes, along with the synaptic cleft —mediate transmission of signals across neurons.^1^, ^2^ Synapses are crucial in establishing and maintaining complex neural connections in the brain.^3^ The development of these connections depends on factors such as the number, variety, and positioning of the synapses. Efficient synaptic function relies on the precise alignment of presynaptic and postsynaptic specializations across a synaptic cleft.^4^ Indeed, electron microscopy has demonstrated that when nerve terminals are isolated through biochemical synaptosome fractionation, the pre‐ and postsynaptic components stay intact and consistently well‐aligned.^5^, ^6^ This implies that specialized molecular mechanisms are required to ensure the proper assembly of these synaptic partners.^7^, ^8^, ^9^, ^10^

Synaptic adhesion molecules (SAMs), which are vital glycoproteins located on neuronal cell surfaces, primarily expressed at synaptic plasma membranes, play an essential role in inducing the formation and assembly of synaptic contacts.^11^, ^12^, ^13^, ^14^ Trans‐SAMs organize synaptic junctions by bridging the synaptic cleft.^15^ SAMs link the pre‐ and postsynaptic compartments while facilitating trans‐synaptic signaling and recognition. These processes are vital for the formation, specification, and plasticity of synapses.^15^, ^16^ An increasing variety of SAMs, including neurexins and neuroligins,^17^ immunoglobulin (Ig) domain proteins‐like synaptic cell adhesion molecule (SynCAM) and neural cell adhesion molecule (NCAM),^12^ receptor phosphotyrosine kinases and phosphatases, as well as various leucine‐rich repeat proteins, have been identified.^16^ These molecules stabilize and maintain the initial connections between neurons and play a major role in synapse maturation, specificity, and functionality, which are essential for optimal neuronal development.^18^, ^19^ Effective synaptic adhesion signaling is essential for regulating neural circuit architecture and brain function.^20^ Disruptions in these pathways are frequently associated with neurodevelopmental disorders (NDDs).^20^ Perturbation of SAMs has been implicated in NDDs.^21^ NDDs like autism, attention deficit disorder, intellectual disabilities, and cerebral palsy are characterized by a spectrum of challenges stemming from atypical brain development. The cause of these disorders is not completely understood, but encompasses gene and environment interactions.^22^, ^23^, ^24^, ^25^, ^26^ Even low levels of exposure to environmental toxins can significantly impact the developing brain, resulting in lasting neurological impairments, particularly during critical periods of growth and maturation.^27^ Metals are widely recognized as prevalent environmental toxicants and have been documented as risk factors for the onset and progression of NDDs.^28^ Numerous studies have linked levels of neurotoxic metals to the etiology of NDDs.^29^, ^30^, ^31^, ^32^, ^33^ This review explores the impact of metal neurotoxicity on SAM signaling and its role in the etiology of NDDs. It provides an overview of SAM signaling and highlights how its disruption may contribute to these disorders.

## 2 OVERVIEW OF SAM SIGNALING

The brain uses action potentials and neurotransmitters to send impulses among billions of synapses in a complex yet highly coordinated process.^34^ According to Piechotta et al.,^35^ synapses are important locations for regulation in brain circuits. They are typified by protein‐protein interactions that collaborate across the synaptic junctions. SAMs are the collective term for these protein complexes that traverse the synaptic cleft.^36^ Neurotransmitter release and postsynaptic receptor ion channel gating play a major role in mediating communication between neurons at synapses. While it is well known that the dysfunction of SAMs causes distortions in neurotransmitter release, it is now appreciated that SAMs that interact in a homo‐ or heterophilic manner across the synaptic cleft can affect communication signals.^37^, ^38^ SAMs are proteins found at every synaptic cleft and have dynamic roles during postnatal and developmental stages. Their functions enable neurons to proliferate and process complex impulse patterns that are the basis of cognition.^1^, ^15^ SAMs nucleate developing synapses, promote synapse maturation, manage synaptic characteristics, and govern synapse elimination.^39^ Due to the ubiquity of SAMs within synapses as main agents in organizing synaptic junctions within pre‐ and postsynaptic neurons, most literature alternatively refers to SAMs as “synaptic organizing molecules”.^40^, ^41^ However, despite the ubiquitous role of SAMs within synapses, it is unlikely that a single “master” SAM is in charge of everything. Instead, a complex ensemble of SAMs and neurotransmitter receptors, alongside cytoplasmic scaffolding proteins, mediate trans‐cellular interactions at diverse synaptic junctions.^39^, ^42^ Apart from connecting pre‐ and postsynaptic neurons via SAMs, they are also known to enable trans‐synaptic recognition and signaling processes crucial for the development, specification, and plasticity of synapses throughout life.^3^, ^15^ The regulation of transmission and plasticity of excitatory synapses is a novel role for SAMs.^43^ According to Sudhof,^39^ the criteria for SAMs are their presence at synapses during different developmental stages, their role in cell‐cell interactions, and evidence showing changes in synapse formation and/or function when these proteins are either lost or overexpressed. In general, SAMs carry out two overlapping tasks: arranging the synaptic assembly (“making synapses”) and defining the features of individual synapses (“shaping synapses”). Sudhof ^39^ claims that SAMs involved in shaping synapses are known to be more than those that make synapses, likely due to the need for diverse synapse properties to be regulated by various types of signals. However, some SAMs perform both functions, while others can only perform one. For instance, unlike SPARC‐like 1 (SPARCL1), a unique, secreted non‐neuronal protein that functions as a synaptogenic factor,^44^ capable of performing both tasks,^45^ neuroligins do not influence synapse numbers but change the properties of synapses, that is, shape synapses.^46^, ^47^ The establishment of chemical synapses within the central nervous system is highly controlled by SAMs and depends on bidirectional communication across the synaptic cleft.^48^

Typically, presynaptic and postsynaptic specializations are positioned at the axon and dendritic spines, respectively. Nonetheless, in certain brain areas, such as the thalamus and olfactory bulb, dendrites themselves can form presynaptic structures, leading to the development of dendrodendritic synapses,^49^, ^50^ while postsynaptic specializations can also be formed on axons. Additionally, all presynaptic specializations use mainly the same molecular machinery to release neurotransmitters. In contrast, postsynaptic specializations vary based on the type of synapse, particularly whether it is excitatory (enhancing signal transmission) or inhibitory (reducing signal transmission), as they involve distinct receptors and associated proteins.^39^, ^51^ Biochemical and ultrastructural studies of synapses suggest that SAMs, which are pre‐ and postsynaptically localized, are critical for the proper assembly and molecular composition of pre‐ and postsynaptic specializations.^52^, ^53^ For instance, neurexin and neuroligin, which both act as calcium‐dependent SAMs, are primarily localized on the presynaptic and postsynaptic neurons, respectively.^54^, ^55^ However, there is evidence suggesting that neurexin may have noncanonical roles at postsynaptic sites under certain conditions or during specific stages of synaptic development and plasticity.^56^

The presynaptic neurexin and its postsynaptic binding partner, neuroligin, make up one of the well‐studied pairings of SAMs.^57^, ^58^ Vertebrates have three neurexin genes (*Nrxn1*, *Nrxn2*, *Nrxn3* in animals; *NRXN1*, *NRXN2*, *NRXN3* in humans),^59^ whereas neuroligins are expressed from four genes in vertebrates (neuroligin‐1 (*NLGN1*) to *NLGN4*).^60^, ^61^ However, primates have been shown to possess nonrecombining variants of *NLGN4* on both X‐ and Y‐chromosomes, with the Y‐chromosomal variant often identified as *NLGN5*.^62^, ^63^ Studies have shown that mice lacking *Nrxn2* display a reduction in excitatory presynaptic transmission and impaired postsynaptic N‐methyl‐d‐aspartate receptor (NMDAR) activity. Conversely, targeted conditional knockouts of *NLGN3* during late development or mutations in neuroligin‐3 (R451C and R704C) significantly impact α‐amino‐3‐hydroxy‐5‐methyl‐4‐isoxazole propionic acid receptor function.^64^, ^65^ The interaction between neurexins and neuroligins to establish a trans‐synaptic complex is facilitated by the sixth laminin G (LG) domain found in α‐neurexin.^66^ While both α‐ and β‐neurexins interact with neuroligins through the same LG domain,^67^ variations in binding affinity have been observed among different neurexin and neuroligin isoforms, primarily due to alternative splicing within their LG domains. These LG domains consist of a lectin‐like β‐sandwich structure that includes a conserved calcium (Ca²⁺)‐binding site located at the variable edge of the domain.^67^, ^68^ Findings show that the splice site 4 (S4) insertion of the LG domain in neurexin‐1 reduces clustering of neuroligin‐1/3/4 and glutamatergic proteins, but not neuroligin‐2 and GABAergic proteins. The S4 insert also lowers neurexin‐1's binding affinity for neuroligins‐1 and‐4.^69^ Iijima et al.^70^ further demonstrated that Src‐associated in mitosis 68 kDa protein (SAM68), an RNA regulator downstream of growth signaling factors, plays an essential role in the activity‐dependent alternative splicing of the neurexin‐1.

**Figure 1:**
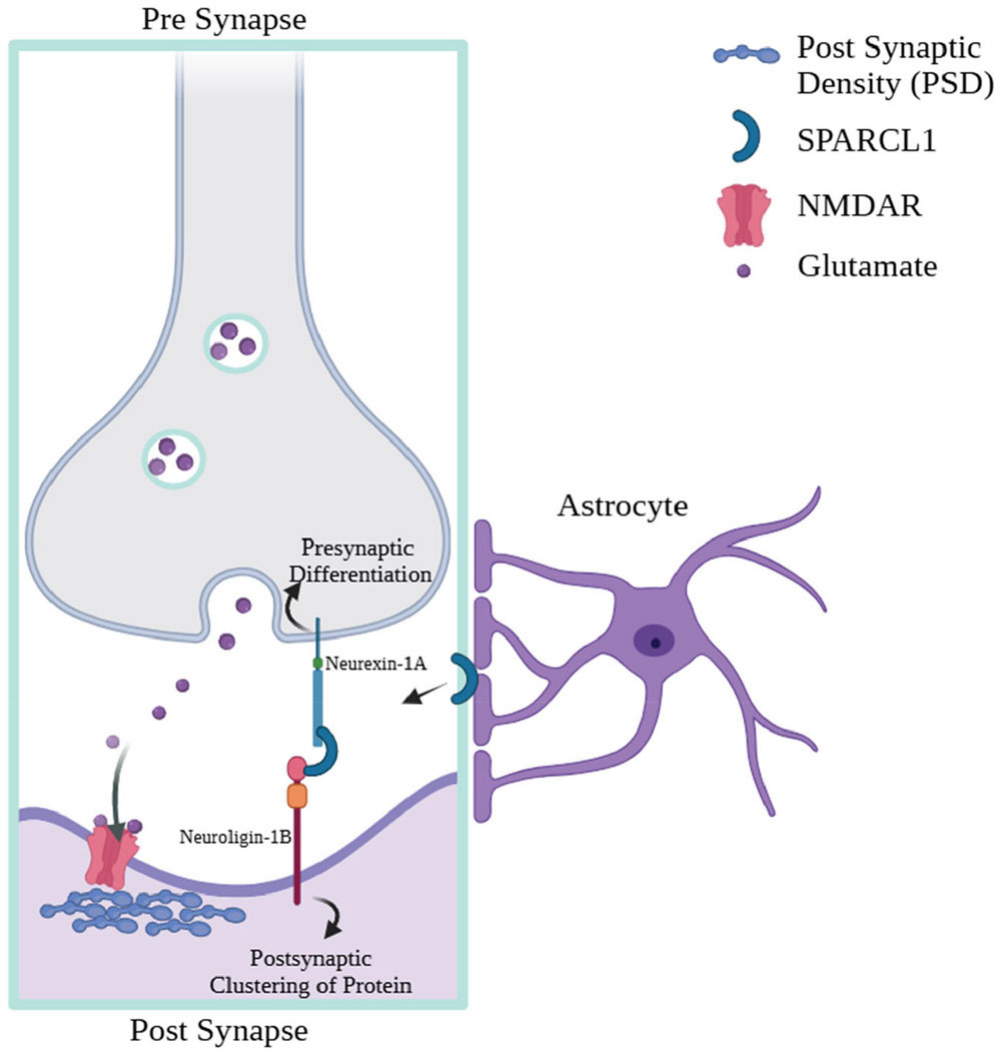
Tripartite synapses indicating astrocyte‐mediated modulation of synapse formation through SPARCL1 signaling. The illustration shows SPARCL1 bridging neurexin‐1A and neuroligin‐1B at the synapse, promoting presynaptic differentiation and postsynaptic clustering of proteins such as NMDARs within the PSD. Abbreviation: SPARCL1, SPARC‐like 1; NMDAR, N‐methyl‐d‐aspartate receptor; PSD, postsynaptic density. [Color figure can be viewed at wileyonlinelibrary.com]

In addition to the pre‐ and post‐synapse, the model of tripartite synapse involving glial cells ^71^, ^72^ and tetrapartite synapse involving glial cells and extracellular matrix ^73^, ^74^ have also been actively involved in synaptic transmission. Growing evidence supports these concepts, highlighting the roles of glial cells and the extracellular matrix in modulating both the structural integrity and functional dynamics of synaptic plasticity. For example, SPARCL1, which is believed to be secreted by end feet of astrocytes, has been shown to facilitate interactions between neurexin‐1A and neuroligin‐1B (which includes splice insert B), a trans‐synaptic pair that typically does not bind directly. This interaction promotes presynaptic differentiation and the clustering of postsynaptic proteins (Figure 1), such as NMDARs, particularly at thalamocortical synapses.^75^ Additionally, perineuronal nets formed by extracellular matrix molecules that are released by neurons and glial cells have been proven to play a significant role in synaptic homeostasis.^76^, ^77^

## 3 SAMS AND NEURODEVELOPMENTAL PERTURBATIONS

### 3.1 Role of SAMs in neurodevelopment

Multiple trans‐synaptic interactions play significant roles in synapse formation and maintenance during an organism's early developmental stage.^10^, ^78^ Different criteria must be fulfilled throughout an animal's lifespan to ensure proper development and maintenance of synaptic networks, such as the functional contribution of various molecules to neurodevelopment. These molecules encompass pre‐, and post‐SAMs.^3^, ^79^ Neurexin and the leukocyte common antigen‐related (LAR)‐type receptor tyrosine phosphatases (LAR‐type PTPRs), for example, are pre‐SAMs that interact with various classes of post‐synaptic cell‐adhesion molecules to facilitate the formation and maintenance of synapses.^78^, ^80^, ^81^ Also, neuroligin, a type of post‐SAMs involved in synaptogenesis, interacts with neurexin to form a heterophilic adhesion complex that contributes to the formation, stability, and functional properties of synaptic connections.^78^, ^82^ During development, neurons migrate to form networks through which they interact. Several SAMs contribute to this neuronal migration (e.g., MAM domain‐containing glycosylphosphatidylinositol anchors and fibronectin leucine‐rich repeat transmembrane proteins) and axonal extension—a phenomenon known as axonal pathfinding.^83^, ^84^ The axons subsequently form synapses on target neurons (or other cells) to construct brain circuits. These molecules use distinct ligands for their various activities, as demonstrated with LAR‐type PTPRs.^85^

Synaptic adhesion proteins form, maintain, and control the tight connection between the presynaptic and postsynaptic terminals.^86^ Various mechanisms exist through which synaptic connections are formed. Understanding how neuronal circuits are built depends on synaptic pruning and synapse synthesis. During neurodevelopment, there is an over‐proliferation of synapses; however, in adolescents, 40%–50% of these synapses are pruned or eliminated.^51^, ^87^ Exemplary instances of embryonic synapse elimination demonstrate that synapses frequently engage in competition, resulting in the disappearance of the “losers” and the dominance of the “winners”; this has been vividly demonstrated for the neuromuscular junction,^88^, ^89^ retinal inputs into the lateral geniculate nucleus.^90^, ^91^

### 3.2 Types of SAMs involved in NDDs

SAMs are classified into superfamilies consisting of integrins, cadherins, immunoglobulins, C‐type lectin‐like domain proteins, and neurexin/neuroligin.^92^, ^93^ These molecules have three distinct conserved domains involved in synaptic interactions: an external domain that communicates with the extracellular matrix, a transmembrane domain that functions within the bilayer, and an intracellular domain that communicates with the cellular cytoskeleton. The extracellular matrix, however, is more involved in synapse formation through mechanisms like docking geometry regulation.^94^ Various genes involved in synaptic processes have been identified and characterized using different animal models.^86^ Studies have demonstrated their crucial contributions to synapse formation since their disruption results in neurological disorders.^10^, ^86^ Over one hundred genes associated with synaptic development have been implicated in the etiology of autism spectrum disorder (ASD).^95^, ^96^ The mutation and consequent disruption of these ASD‐associated genes result in a range of behavioral anomalies. Examples of these genes include: discs large MAGUK scaffold protein 4 (*DLG4*), *NRXN*, *NLGN*, and SH3 and multiple ankyrin repeat domains protein (*SHANK*) genes. Variations in the expression of these genes have been identified to be risk factors for various neurodevelopmental, neurological, and neuropsychiatric disorders, including ASD.^97^

#### 3.2.1 Neurexin/neuroligin

Neurexin is a presynaptic neuronal adhesion molecule essential for synapse formation, function,^98^, ^99^ and plasticity.^100^ Their synaptic function depends on binding to neuroligins and forming complexes in the synaptic cleft.^101^ This interaction is essential for the development, maintenance, and normal functioning of synapses. Neurexin expression and alternative splicing are regulated in a time and region‐specific manner within the brain, resulting in each neuronal subtype exhibiting a distinct pattern of neurexin splice variants.^102^, ^103^ Neurexins interact with a broad range of structurally diverse extracellular ligands, including secreted proteins like cerebellins and neurexophilins, transmembrane proteins such as neuroligins, α‐dystroglycan, leucine‐rich‐repeat transmembrane neuronal proteins, and calsyntenin‐3, as well as receptor‐type molecules like GABA receptors and latrophilins, enabling them to regulate distinct signaling pathways.^104^ These ligand interactions are finely tuned by alternative splicing of neurexins, which shapes their binding specificity and functional roles in synaptic communication.^81^, ^105^ Furthermore, neurexins associate in cis with LAR‐type PTPRs, a process that may be facilitated by the addition of heparan sulfate chains during posttranslational modification.^106^, ^107^ The intracellular C‐terminal domain of neurexins associates with key presynaptic scaffold proteins, including calcium/calmodulin‐dependent serine protein kinase (CASK), Mints, and protein 4.1, which collectively anchor neurexins to the presynaptic apparatus and contribute to synaptic organization.^58^, ^108^ Conversely, on the postsynaptic membrane, neuroligins' intracellular C‐terminus associates with the post‐synaptic density‐95 (PSD‐95), disks‐large and zonula occludens‐1 (PDZ) domain of PSD‐95, a scaffold protein that facilitates the assembly of receptor complexes and signaling proteins.^109^, ^110^, ^111^ The extracellular globular domain of neuroligins, which resembles acetylcholinesterase, facilitates their activity‐dependent interaction with presynaptic neurexins.^112^, ^113^

Perturbed interactions between neurexin*/*neuroligin complexes could lead to abnormal synaptic functioning, and consequently, disrupted neurodevelopment.^78^, ^114^, ^115^, ^116^ Numerous environmental factors have the potential to alter *NRXN/NLGN* families of genes expression, this could be beneficial or detrimental. Dysregulation in the *NRXN* genes ^117^ and the neurexin‐neuroligin binding, have recently gained pathological interest as both families of genes are linked to various neuropsychiatric disorders including autism.^118^ Although only three *NRXNs* exist in the human genome, thousands of neurexin isoforms can be generated through the use of two alternative promoters and alternative splicing.^119^, ^120^ Genetic alterations in the neurexin family (*NRXN1, NRXN2*, and *NRXN3*) have also been consistently observed in individuals with ASD.^120^ Armstong and colleagues examined the behavioral effects of the *Nrxn1α* deletion in NDDs.^121^ Deletion of this gene could be associated with autism, schizophrenia, and mental retardation. Prior examinations using *Nrxn1α* knockout (KO) mice as a model for these conditions have demonstrated deficits in synaptic transmission, but have not shown abnormalities in social behavior, which is one of the main indications of autism. Their findings revealed that the homozygous *Nrxn1α* KO mice showed altered social approaches, decreased social investigation, and decreased locomotor activity in novel surroundings. In addition, aggressive behaviors were observed in male *Nrxn1α* KO mice. The results indicate that the abnormalities observed in social behaviors may be caused by deletions within *NRXN1* and that the *Nrxn1α* KO mice serve as a valuable model for human NDDs.^122^ Further investigation addressed how *Nrxn1α* deletion affected behavior at various developmental stages, demonstrating higher levels of aggression and social deficits in male and juvenile *Nrxn1α* knock‐out mice.^121^

There are five *NLGNs* in the mammalian genome: *NLGN1*, *NLGN2*, *NLGN3*, *NLGN4X*, and *NLGN4Y*. On the X chromosome are *NLGN3* and *NLGN4X*, while on the Y chromosome is *NLGN4Y* (also called *NLGN*5).^17^, ^123^, ^124^ Only rodents express the following four *NLGNs*: *Nlgn1*, *Nlgn2*, *Nlgn3*, and *Nlgn4*. Except for *Nlgn4*, mammalian protein sequences are mostly conserved. Research indicates that the fully developed proteins encoded by these genes are predominantly localized at specific synapses^125^: *NLGN1* is primarily localized at glutamatergic excitatory synapses^126^; *NLGN2* is predominantly localized at GABAergic inhibitory synapses^127^; *NLGN3* is specifically associated with both glutamatergic and GABAergic synapses^128^; *NLGN4X* is concentrated at excitatory synapses.^125^, ^129^ It has been demonstrated that disruption of the *NLGN1* gene causes synapse dysfunction and behavioral abnormalities.^130^ Mutations in *NLGN* genes, primarily located in the extracellular domain and involving missense or heterozygous variants, have been linked to ASD.^130^, ^131^, ^132^, ^133^, ^134^ *In vitro* models of the pathogenic mutations result in diminished contact with presynaptic partners, trafficking to the plasma membrane, or faulty folding of proteins.^135^, ^136^, ^137^ Studies have shown the association between mutation in *NLGN* genes and the pathogenesis of autism and other neurodevelopmental and neuropsychiatric disorders.^130^, ^138^, ^139^ Most mutations correspond to the extracellular domain of the proteins.^125^ The majority of these mutations are present in *NLGN4X* and *NLGN3*, the two X‐linked *NLGN* genes.^115^ *NLGN4X* is most strongly linked to autism; several missense and frameshift variants have been found, with the majority assumed to impair protein function seriously.^125^ A Swedish family with two affected siblings was the first to demonstrate this association between *NLGNs* and autism. One of the siblings had a *de novo* missense mutation in the coding area of *NLGN3*, and the other in *NLGN4X*.^140^ Kopp et al.^141^ also reported that a paternally inherited microdeletion of *NLGN4X* is a genetic cause of ASD in a female. In addition, Trobiani et al.^125^ also demonstrated that autism‐related *NLGN* mutations are proposed to result in endoplasmic reticulum (ER) retention and the activation of ER stress pathways. Tabuchi et al.^21^ investigated the role of a *Nlgn3* gene mutation in mice's inhibitory synaptic transmission in autism. Gene targeting was used by the researchers to introduce the R451C‐substitution into the endogenous *Nlgn3* gene in mice, resulting in R451C knock‐in (KI) animals. Mice with the R451C mutation had better spatial learning ability but worse social relations. Interestingly, there was no discernible impact on excitatory synapses, and these behavioral alterations were correlated with an increase in inhibitory synaptic transmission. On the other hand, no similar alterations were observed upon deletion of *Nlgn3*, suggesting that the R451C‐substitution is a gain‐of‐function mutation. The R451C KI mice may be a valuable model for researching behaviors associated with autism, according to these studies, which also imply that enhanced inhibitory synaptic transmission may lead to ASD in humans. The R451C‐substitution, according to the authors, decreases social behaviors while specifically improving spatial learning capacities. It also enhances inhibitory synaptic transmission without impacting excitatory synaptic transmission.

#### 3.2.2 Cadherins

Cadherins are present in both pre‐and post‐synaptic neurons. There are roughly 20 variations of cadherins expressed in different brain regions.^142^ It is assumed that the specificity of synapse construction results from the matching of the relevant cadherin to the target cell contact.^143^ Cadherins are risk genes for aggression, depression, substance misuse, ASD, and attention‐deficit hyperactivity disorder (ADHD), among other associated neurodevelopmental and psychiatric disorders.^144^ Cadherin‐2 (CDH2) is a cell adhesion molecule involved in brain development with important roles in neurulation, differentiation, migration, axon guidance, synaptogenesis, and synaptic maintenance. Key members of the cadherin superfamily include classical cadherins, protocadherins, and a distinct group known as cadherin‐like neuronal receptors. These proteins, characterized by a single transmembrane domain, mediate robust calcium‐dependent cell–cell adhesion through the first of their five or six tandem extracellular repeat domains.^145^, ^146^, ^147^, ^148^

A mutation in the *CDH2* gene responsible for this protein has been shown to contribute to the pathogenesis of agenesis of corpus callosum, axon pathfinding, cardiac, ocular, and genital defects (ACOG)‐syndrome, ASD, and ADHD.^149^, ^150^, ^151^ Delays in development, intellectual impairment, and abnormalities in axonal pathfinding are among the multiple symptom combinations caused by a mutation in *CDH2*.^152^ These symptoms have been observed in experimental models including mice ^153^, ^154^ and other vertebrates.^155^ The pathogenesis of ASD ^151^ and obsessive‐compulsive disorder has also been linked to, and suggested to be influenced by, the *CDH2* gene.^156^ A study examined the impact of *CDH2* on synapse formation.^143^ The study showed that *CDH2* facilitated synapse differentiation at the presynaptic terminal by its high affinity for joining other synaptic components. In the same study, similar molecules like *CDH4* and *CDH6* were found to replace cadherins, thus demonstrating their functional redundancy. This indicated that cadherins initiate the formation of new synapses and facilitate the upkeep of already‐formed synapses. By manipulating *CDH10*, it was shown that it is important for controlling the ratio of excitatory to inhibitory synapses on dendrites. Overall, abnormalities in the function of cadherins within the cell may cause neurological effects, including autism.^157^ Another study examined the significance of *CDH9* in relation to the formation of hippocampal synapses, noting that *CDH9* significantly influenced the CA3/DG synaptogenic complex, a crucial synaptogenic complex that bidirectionally regulated DG‐CA3 synapse development. The size and quantity of synapses that the CA3/DG complex formed decreased as a result of *CDH9* downregulation. Defects in the synapse's development were also caused by the total lack of *CDH9*.^158^

#### 3.2.3 Integrins

Various neurological disorders can only be prevented by maintaining synaptic integrity. Studies have been conducted on the potential influence of integrins, another subclass of the cell adhesion molecule family, on synapse function concerning NDDs like autism and schizophrenia.^159^ Rather than adhering to other cells, integrins adhere to the extracellular matrix. Integrins, heterodimeric receptors composed of non‐covalently linked *α* and *β* subunits, mediate the binding of extracellular matrix proteins and transmit signals to intracellular pathways and cytoskeletal structures.^160^ Through adaptor proteins such as talin, α‐actinin, and vinculin, they connect to the actin cytoskeleton and interact with various cytoplasmic signaling molecules.^161^ Target recognition, synaptogenesis, synaptic plasticity, brain nucleus formation, neurite growth, and maintenance, are all processes through which integrins regulate neuronal connectivity.^159^ Research has shown that dysfunctional pathways, such as dysregulated serotonin and impaired T‐cell physiology, led to aberrant integrin activity. Serotonin system (5‐HT) within the presynaptic domain has been shown to be altered by the Leu33Pro polymorphism for integrin beta 3 (ITBG3), an integrin within the 5‐HT that is frequently detected in ASD patients. This alteration is linked to anxiety, repetitive behavior, and social behavioral deficits observed in ASD.^162^ Overall, these demonstrate how crucial integrins are to establishing and maintaining synapses and how disruptions to their signaling may result in NDDs.

#### 3.2.4 Ephrin and erythropoietin‐producing hepatocellular (Eph) receptor

Eph receptor belongs to the family of receptor tyrosine kinases and are classified into EphA and EphB. Eph receptors bind with Ephrin ligands. Eph receptors are transmembrane proteins characterized by highly conserved domains on both their extracellular and intracellular regions. The extracellular portion contains an N‐terminal ligand‐binding domain, followed by a cysteine‐rich segment and a pair of fibronectin type III repeats.^163^ Eph receptors can form both homodimers and heterodimers through multiple mechanisms. Direct interactions are mediated by structural elements such as the extracellular cysteine‐rich domain, fibronectin type III repeats, and the intracellular SAM domain. Alternatively, dimerization may occur indirectly through the recruitment of PDZ domain‐containing adaptor proteins.^164^ Due to their unique signaling capacity, Eph‐ephrin bindings can have the ability to function bidirectionally in a contact‐mediated fashion between neurons.^165^ Their bindings play a diverse functional role in the brain, guiding axons during neurogenesis,^166^ regulating synaptogenesis and neuroplasticity in adults.^167^ This bidirectional binding also plays an important role in enhancing presynaptic differentiation.^165^ Numerous studies have linked perturbations in ephrin binding to NDDs.^168^, ^169^ *Ephrin‐A2* knocked mice exhibit normal hippocampus learning and memory, but their ability to adapt behaviorally in a reversal learning/set shifting test is compromised.^170^ This behavior closely mirrors the core symptoms of ASDs observed in both humans and animal models.^171^

## 4 UPTAKE OF METALS IN THE BRAIN AND THEIR EFFECTS ON NEURONS, GLIA, AND SYNAPSES

Brain can accumulate neurotoxic metals through various pathways. Distinguishing between metals essential for biological functions and those exerting toxic effects is often challenging. Micronutrients such as cobalt (Co), copper (Cu), iron (Fe), manganese (Mn), molybdenum (Mo), and zinc (Zn) have the potential to disrupt normal physiological processes when present in excessive amounts.^172^ Therefore, maintaining a precise balance between deficiency and toxicity is particularly crucial in sensitive tissues, such as the brain. These essential metals utilize specific transport mechanisms to cross the blood‐brain barrier (BBB) and enter cells.^173^, ^174^ These mechanisms involve transporters such as natural resistance‐associated macrophage protein 2/divalent metal transporter 1 (NRAMP2/DMT‐1) and high‐affinity copper transporter 1 (CTR1).^175^ Due to limited selectivity, the same transport proteins that mediate the uptake of essential metals can also transport nonessential metals sharing similar ionic size, charge, or chemical properties. For instance, cadmium (Cd²⁺) can be imported into cells via Zrt/Irt‐like protein transporters, which typically mediate the uptake of Zn²⁺ from the extracellular environment into the cytoplasm.^176^ Neurotoxic metals, particularly heavy metals, can access the brain by crossing the BBB through several pathways. These include receptor‐ or carrier‐mediated transport, passive diffusion, and movement through intercellular gaps in the brain's endothelial cells.^177^ While some neurotoxic metals penetrate the brain by crossing the BBB, others bypass this barrier and enter directly through the olfactory routes. Primary olfactory neurons within the olfactory epithelium have dendrites that interact with the nasal lumen and axons that project to the olfactory bulbs of the brain. Substances such as metals that interact with the olfactory epithelium can be absorbed by primary olfactory neurons and subsequently transferred to the olfactory bulbs, with the potential to spread further into other regions of the brain.^178^, ^179^, ^180^ After entering the brain, certain heavy metals tend to build up within neurons and glial cells in specific regions like the hippocampus and frontal cortex, areas essential for learning, memory, and executive functions. The accumulation of these metals in such critical regions can disrupt synaptic activity, ultimately leading to cognitive impairments.^181^, ^182^ Neurotoxic metals, upon entering the brain, disturb the typical physiological activities of neurons and glial cells.^33^ For example, astrocytes play a crucial role in maintaining ion balance within the interstitial fluid through a diverse range of membrane‐bound transporters.^183^, ^184^ Specifically, astroglia transporters help clear excess heavy metals from the brain tissue, thereby safeguarding neurons from metal‐induced toxicity.^185^, ^186^ However, the build‐up of heavy metals impairs astrocyte function and their mitochondrial inner membrane potential,^187^ disrupting their essential homeostatic and neuroprotective processes,^188^ particularly those involved in glutamate‐glutamine cycling and antioxidant defense mechanisms.^187^ Neurotoxic metals also interfere with presynaptic neurotransmitter release by binding to specific molecular targets within this pathway.^189^ In some cases, a single metal can simultaneously affect multiple targets, amplifying its disruptive effects. For instance, aluminum interferes with neuronal signaling by blocking voltage‐gated calcium channels, reducing the functional activity of calmodulin (CaM), and inhibiting Ca²⁺‐ATPase, thereby impairing calcium homeostasis.^190^, ^191^, ^192^ Moreover, Cd diminishes the currents through voltage‐activated calcium channels, thereby altering intracellular calcium levels. This disruption affects the activation of CaM and disturbs calcium‐dependent intracellular signaling pathways. Cd exposure also leads to a reduction in excitatory neurotransmitters release such as aspartate and glutamate, while simultaneously enhancing the release of inhibitory neurotransmitters like GABA and glycine.^193^, ^194^ Postsynaptically, neurotoxic metals can disrupt neurotransmitter receptor function through multiple mechanisms.^195^ These include altering the gene or protein expression of the receptors,^196^ indirectly impairing their activity through the generation of reactive oxygen species,^197^ or directly competing with essential physiological ions at receptor binding sites.^192^ For example, arsenic (As) exposure has been shown to alter the expression of NMDA and AMPA receptors in specific brain regions of animal models.^198^ In contrast, Cd exposure disrupts the function of several neurotransmitter receptors in the brain, including those for glutamate, acetylcholine, GABA, and dopamine. Cd also interferes with muscarinic acetylcholine receptors. Specifically, it induces cell death in primary cholinergic neurons of the basal forebrain by suppressing the activity of the M1 muscarinic receptor.^192^, ^199^, ^200^

## 5 METAL NEUROTOXICITY AND NDDS

Growing industrialization has led to an increase in environmental toxicants, such as metals. Though some are deemed essential, metals are widely recognized as prevalent environmental toxicants and have been documented to contribute to the occurrence of NDDs.^28^ Continuous exposure to metals, especially heavy metals, from diverse environmental sources, such as food, air, and water, can be detrimental to human health and result in neurotoxicity. Given that heavy metal exposure during the developmental period can cause neurological perturbations, developmental delays, learning difficulties, and behavioral deficits, they are known to be neurodevelopmental toxicants.^201^ Even low‐level exposure to metals, especially during critical developmental stages, can profoundly affect health, leading to enduring neurological deficits.^27^ Excessive environmental exposure to heavy metals has been intricately associated with the genesis of NDDs, particularly autism.^26^ Elevated blood levels of these metals have been associated with a higher prevalence of autism in multiple reports.^202^ Prenatal or early‐life exposure to heavy metals like Mn, lead (Pb), mercury (Hg), Cd, and nickel (Ni) has been linked to the onset of NDDs. The Agency for Toxic Substances and Disease Registry (ATSDR) has identified metals like Pb, Hg, As, and Cd, as potential harmful substances that have been implicated in perturbations in developmental processes and neurological dysfunctions.^31^, ^32^, ^203^, ^204^ For instance, children with NDDs such as autism and ADHD have higher blood levels of Ni.^205^ Nayak et al.^206^ investigated the involvement of Cd, Ni, and As in ADHD pathogenesis by measuring heavy metal concentrations in blood and urine samples. Their findings revealed a correlation between increased levels of Pb, Cd, and Ni and ADHD, indicating that these metals may contribute to the development of the disorder. Early postnatal Mn exposure has also been reported to trigger prolonged attentional disruption in adult rats, although learning and impulse control remain intact.^207^ Similarly, Schneider et al.^208^ also demonstrated that Mn exposure induced attention dysfunction in nonhuman primates. Moreover, multiple epidemiological studies that have examined the role of Mn exposure in the etiology of NDDs, specifically ASD, have reported varying results. In a recent study that investigated the level of essential elements, including Mn in the hair of 227 children within the age range of 3–14 years (120 controls and 107 with ASD), Ouisselsat et al.^209^ reported that there is a significant reduction in the Mn level in the hair sample of ASD children compare to the control group. Conversely, Ma et al.^210^ found no significant difference in serum Mn concentrations between individuals with ASD and control subjects. Perturbation in doperminergic system has been linked to autistic behavior.^211^ Mn has been repeatedly linked to dopaminergic dysfunction causing structural, functional, and neurochemical alterations in the dopaminergic system.^212^, ^213^, ^214^ This corroborates that Mn overexposure plays a role in the pathophysiology of ASD. In some cases, deficiencies in essential metals have also been linked to the development of NDDs. For instance, iron deficiency—despite its critical role in oxygen transport, energy production, cell proliferation, and DNA synthesis ^215^—has been implicated in NDD etiology.^216^, ^217^ Chen et al.^218^ indicated in their meta‐analysis study that upregulation in serum transferrin level might be linked to a high risk of autism. Similarly, Latif et al.^219^ associated iron deficiency as a risk factor of autism. In another context, excessive iron intake during development has been shown to disrupt the regulation of brain iron and cause iron‐associated neurodegeneration in later life.^220^

## 6 SAMS SIGNALING AND METAL NEUROTOXICITY IN NEURODEVELOPMENTAL PATHOGENESIS

Given that synapses serve as the fundamental units for information processing in the brain, it is plausible that synaptic dysfunction underlies a wide array of brain disorders.^221^, ^222^ A thorough grasp of synaptic function is essential for understanding how the brain processes thoughts, feelings, behavior,^223^ and what processes are perturbed in NDDs. Given the role of SAMs in determining synaptic functions such as maturation, remodeling, and formation, abnormal synaptic plasticity and failure to establish proper synaptic contacts might represent mechanisms underlying the risk of NDDs. This highlights the essential role of SAMs in sustaining healthy brain function and their potential contribution to the development of various NDDs.

**Figure 2:**
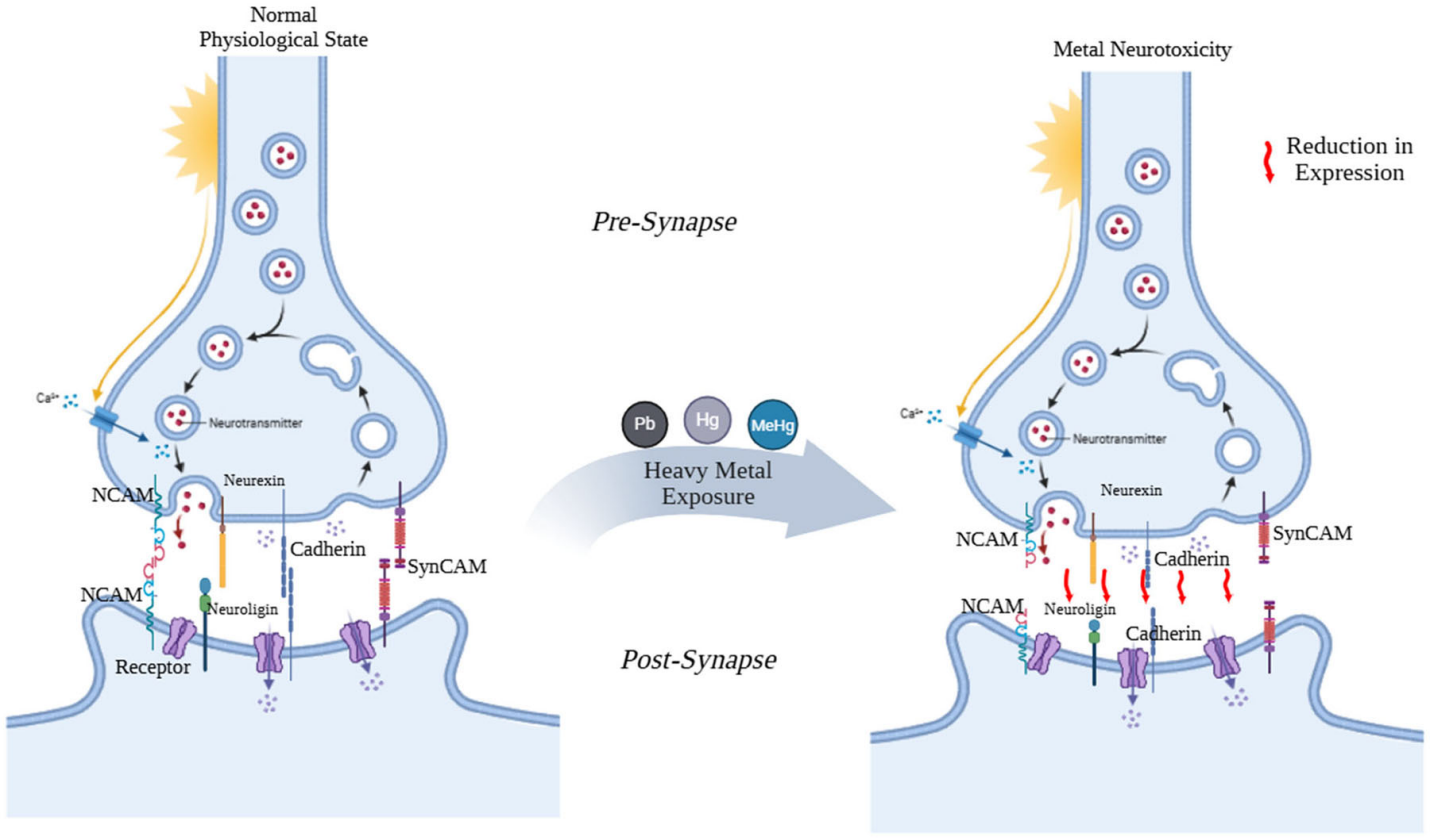
Interaction between early life exposure to neurotoxic metals and synaptic adhesion molecules. Exposure to those metals leads to disruptions in the expression in synaptic adhesion molecules. Hg, mercury; MeHg, methylmercury; NCAM, neural cell adhesion molecule; Pb, lead; SynCAM, synaptic cell adhesion molecule. [Color figure can be viewed at wileyonlinelibrary.com]

Early exposure to neurotoxic metals can result in lasting effects on offspring's health, including significant long‐term impacts on neurodevelopmental outcomes.^224^ The prevalence of neurotoxic metals has increased as a result of industrialization.^225^ The molecular mechanism behind how these metals specifically influence SAMs to cause NDDs is not fully understood. Interaction between early life exposure to these neurotoxic metals and SAMs in the pathogenesis of NDDs is likely. As shown in Table 1, metals can disrupt the functions of SAMs. For example, metals can interact with SAMs through mechanisms that involve the modification of their gene and/or protein expression (Figure 2). A recent study conducted by Gunderson et al.^226^ stated the role of neuroligin in the developmental neuromuscular toxicity caused by methylmercury (MeHg). This study is fundamental in comprehending the connection between SAMs, metal exposure, and neuromuscular impairment by researching how early life exposure to MeHg influences the expression of neuroligin and neurexin, which chronically lowers their expression during the early stages of adulthood. This study underlines the relationship between metal‐induced neurotoxicity and alteration in SAM signaling. Hunter et al.^227^ reported that mutations in the neuroligin genes in *C. elegans* result in hypersensitivity to Hg and Cu, indicating a potential link between genetic abnormalities in genes encoding synaptic adhesion proteins like neuroligin and perturbation in synaptic formation and function. Such genetic defects may increase sensitivity to neurotoxic metals, such as Hg and Cu, and could be associated with the development of ASD. Tu et al.^228^ reported upon developmental exposure to Pb, Mn, and Cd decreased expression of Nrxn2aa and Nrxn2ab in the central nervous system of zebrafish, in turn, affecting their neurobehavior by causing a significant decrease in larval swim distance and velocity. Luo et al.^229^ found that maternal exposure to arsenite altered neurobehavior in rat offspring by lengthening the time it takes for them to complete reflex responses such as surface righting, negative geotaxis, and cliff avoidance. They also found that the expression of NCAM and polysialylated form (PSA)‐NCAM in the rat offspring's hippocampal regions was upregulated in a dose‐dependent manner. Polysialation of NCAM is essential for NCAM‐mediated cellular contacts and functions^235^; nevertheless, the expression of PSA‐NCAM is strictly regulated, and any modifications or perturbations might disrupt brain development,^236^ while prolonged PSA expression leads to improper synaptic structure. Therefore, elevated expression of PSA‐NCAM and NCAM during the critical phase of brain development may explain the neurodevelopmental impairments observed in children after As exposure.^229^ Developmental exposure to MeHg disrupted the expression of NCAM polysialylation in rats. This disruption may interfere with the typical formation of neuronal contacts, potentially contributing to the behavioral and morphological disturbances observed after MeHg exposure.^230^ Marchand et al.^231^ reported that early Cd exposure impairs NCAM expression in *Xenopus laevis* tadpoles and altered their behavior by decreasing their maximum swimming speed and increasing their swimming distance traveled relative to unexposed controls. Studies have also demonstrated that MeHg modified the expression of ephrin in the P19 embryonal carcinoma cell line; this alteration may be responsible for the occurrence of path‐finding mistakes and cognitive impairments.^232^, ^237^ Early exposure to Pb during neurodevelopmental periods in herring gulls disrupted the expression of NCAM and N‐cadherin.^233^ This early lead exposure has also been associated with neurobehavioral deficits, including dissociation,^234^ a symptom frequently observed in individuals with developmental disorders such as autism.^238^ There is additional evidence that learning and memory deficits that occur after developmental low‐Pb exposure are related to disruption in the expression and functionality of NCAM.^239^, ^240^

**Table 1:** Summary of how early exposure to metals disrupts the expression of synaptic adhesion molecules and their effects on behavior across different research models.

SAM (model used)	Metal	Stages of exposure	Key effects on SAM	Impact on the behavior	References
Neuroligin and Neurexin (*Drosophila melanogaster)*	MeHg	larvae (L1)	Early life exposure to MeHg chronically lowers nlg and nrx expression during the early stages of adulthood.	Reduction in flight ability	[226]
Neuroligin (*C elegans*)	Hg and Cu	Young adult	Mutations in the neuroligin genes result in hypersensitivity to metals.	*Nlgn1*mutants exhibit specific chemosensory and behavioral deficits while responding normally to most chemical cues.	[227]
Neurexin (Zebrafish)	Pb, Mn, and Cd	Embryonic stage	Decreased expression of Nrxn2aa and Nrxn2ab in the central nervous system	Disturbance in neurobehavior resulting in a substantial reduction in larval swimming distance and velocity	[228]
NCAM and PSA‐NCAM (rat offspring)	As	Four‐month‐old pregnant rat (Day 0 of gestation – post natal day (PND) 21); Offspring (PND 21)	Upregulate the expression of NCAM and PSA‐NCAM	Significantly extended the duration required for rat offspring to execute reflexive reactions, including surface righting, negative geotaxis, and cliff avoidance.	[229]
NCAM (rat; cerebellum)	MeHg	PND 3	Early exposure to MeHg disrupts the expression of NCAM polysialylation	Nil	[230]
NCAM (*Xenopus laevis* tadpoles)	Cd	Embryo‐Tadpoles (stage 45)	Downregulate the expression of NCAM	A decrease in maximal swimming speed and an increase in swimming distance and cardiovascular rhythm	[231]
Ephs‐ A2, A3, B3 and B6 and ephrins A5, A6, B1 and B2 (P19 embryonal carcinoma cell line)	MeHg	Neuronal cell culture	Disruption in the expression of Eph receptor mRNA	Nil	[232]
NCAM and N‐cadherin (Herring gull)	Pb	Post‐hatching day 2	Early exposure disrupts the expression of NCAM and N‐cadherin	Influence the neurobehavioral development	[233, 234]

Abbreviations: SAM, synaptic adhesion molecule; MeHg, methylmercury; nlg, neuroligin; nrx, neurexin; Nlgn1, neuroligin‐1; Nrxn2aa, neurexin 2aα; Nrxn2ab, neurexin 2aβ; NCAM, neural cell adhesion molecule; PSA‐NCAM, polysialylated neural cell adhesion molecule; PND, postnatal day; Ephs, ephrin tyrosine kinase receptors; Ephrins, ephrin ligands; mRNA, messenger ribonucleic acid; N‐cadherin, neural cadherin; Pb, lead; Mn, manganese; Cd, cadmium; Hg, mercury; Cu, copper; As, arsenic.

## 7 CONCLUSION AND FUTURE DIRECTION

SAMs are essential for the formation, maturation, function, plasticity, and assembly of synaptic connections, all of which are critical for normal neurodevelopment. As highlighted in this timely review, excessive exposure to neurotoxic metals during development could disrupt the developing brain and potentially lead to NDDs. Overexposure to these metals can also interfere with SAM signaling, and disruptions in these pathways are frequently linked to NDDs. Perturbation in SAMs have been implicated in the etiology of these disorders.

Neurotoxic metals employ various mechanisms to disturb the normal process of neurodevelopment. Here we posit a possible interconnection between metal neurotoxicity and SAM perturbation in the etiopathogenesis of NDDs. Metal neurotoxicity influences the expression of SAMs, which could subsequently lead to neurobehavioral dysfunction. However, the current literature requires additional evidence to fully elucidate this mechanism. Therefore, more focused research is needed to better characterize the relationship between metal exposure, SAM perturbations, and NDDs.

## AUTHOR CONTRIBUTIONS

Toheeb O. Oyerinde and Omamuyovwi M. Ijomone conceptualized the study. Toheeb O. Oyerinde, Victor E. Anadu, Tobiloba S. Olajide, and Ileje I. Ukwubile wrote the original draft. Olayemi K. Ijomone, Kingsley A. Iteire, Michael Aschner, and Omamuyovwi M. Ijomone revised and edited the manuscript. All authors read and approved the final version of this manuscript.

## CONFLICT OF INTEREST STATEMENT

The authors declare no conflict of interest. The content is solely the responsibility of the authors and does not necessarily represent the official views of the Fogarty International Center (FIC) nor the National Institutes of Health (NIH).

## ETHICS STATEMENT

Since it involved no primary research (human subjects, animal experiments, etc.), it required no ethical approval. All sources are appropriately cited, and the authors declare no ethical conflicts.

## Data Availability

Not applicable as no new data were generated in this review.

## References

[1] Harris KP , Littleton JT . Transmission, development, and plasticity of synapses. Genetics. 2015;201(2):345‐375. 10.1534/genetics.115.176529 26447126 PMC4596655

[2] Südhof TC . Towards an understanding of synapse formation. Neuron. 2018;100(2):276‐293. 10.1016/j.neuron.2018.09.040 30359597 PMC6226307

[3] Rudenko G . Dynamic control of synaptic adhesion and organizing molecules in synaptic plasticity. Neural Plast. 2017;2017(1):6526151. 10.1155/2017/6526151 28255461 PMC5307005

[4] Biederer T , Kaeser PS , Blanpied TA . Transcellular nanoalignment of synaptic function. Neuron. 2017;96(3):680‐696. 10.1016/j.neuron.2017.10.006 29096080 PMC5777221

[5] Lisman JE , Harris KM . Quantal analysis and synaptic anatomy‐‐integrating two views of hippocampal plasticity. Trends Neurosci. 1993;16(4):141‐147. 10.1016/0166-2236(93)90122-3 7682347

[6] Evans GJO . The synaptosome as a model system for studying synaptic physiology. Cold Spring Harbor Protocols. 2015;2015(5):pdb.top074450. 10.1101/pdb.top074450 25934942

[7] El‐Husseini A . Adhesion Molecules at the Synapse. Structural and Functional Organization Of The Synapse. Springer; 2008:173‐204.

[8] Scheiffele P . Cell‐cell signaling during synapse formation in the CNS. Annu Rev Neurosci. 2003;26(1):485‐508. 10.1146/annurev.neuro.26.043002.094940 12626697

[9] Wang M , Fan J , Shao Z . Cellular and molecular mechanisms underlying synaptic subcellular specificity. Brain Sci. 2024;14(2):155. 10.3390/brainsci14020155 38391729 PMC10886843

[10] Batool S , Raza H , Zaidi J , Riaz S , Hasan S , Syed NI . Synapse formation: from cellular and molecular mechanisms to neurodevelopmental and neurodegenerative disorders. J Neurophysiol. 2019;121(4):1381‐1397. 10.1152/jn.00833.2018 30759043

[11] Scheiffele P , Fan J , Choih J , Fetter R , Serafini T . Neuroligin expressed in nonneuronal cells triggers presynaptic development in contacting axons. Cell. 2000;101(6):657‐669. 10.1016/s0092-8674(00)80877-6 10892652

[12] Biederer T , Sara Y , Mozhayeva M , et al. SynCAM, a synaptic adhesion molecule that drives synapse assembly. Science. 2002;297(5586):1525‐1531. 10.1126/science.1072356 12202822

[13] Verpoort B , de Wit J . Cell adhesion molecule signaling at the synapse: beyond the scaffold. Cold Spring Harbor Perspect Biol. 2024;16(5):a041501. 10.1101/cshperspect.a041501 PMC11065171 38316556

[14] Bogaciu CA , Rizzoli SO . Membrane trafficking of synaptic adhesion molecules. J Physiol. 2024;603(20):5921‐5934. 10.1113/JP286401 39322997 PMC12559978

[15] Dalva MB , McClelland AC , Kayser MS . Cell adhesion molecules: signalling functions at the synapse. Nat Rev Neurosci. 2007;8(3):206‐220. 10.1038/nrn2075 17299456 PMC4756920

[16] Missler M , Sudhof TC , Biederer T . Synaptic cell adhesion. Cold Spring Harbor Perspect Biol. 2012;4(4):a005694. 10.1101/cshperspect.a005694 PMC3312681 22278667

[17] Südhof TC . Neuroligins and neurexins link synaptic function to cognitive disease. Nature. 2008;455(7215):903‐911. 10.1038/nature07456 18923512 PMC2673233

[18] Körber N , Stein V . In vivo imaging demonstrates dendritic spine stabilization by SynCAM 1. Sci Rep. 2016;6(1):24241. 10.1038/srep24241 27053173 PMC4823656

[19] Morimura N , Yasuda H , Yamaguchi K , et al. Autism‐like behaviours and enhanced memory formation and synaptic plasticity in Lrfn2/SALM1‐deficient mice. Nat Commun. 2017;8(1):15800. 10.1038/ncomms15800 28604739 PMC5472790

[20] Kim HY , Um JW , Ko J . Proper synaptic adhesion signaling in the control of neural circuit architecture and brain function. Prog Neurobiol. 2021;200:101983. 10.1016/j.pneurobio.2020.101983 33422662

[21] Tabuchi K , Blundell J , Etherton MR , et al. A neuroligin‐3 mutation implicated in autism increases inhibitory synaptic transmission in mice. Science. 2007;318(5847):71‐76. 10.1126/science.1146221 17823315 PMC3235367

[22] Cheroni C , Caporale N , Testa G . Autism spectrum disorder at the crossroad between genes and environment: contributions, convergences, and interactions in ASD developmental pathophysiology. Mol Autism. 2020;11(1):69. 10.1186/s13229-020-00370-1 32912338 PMC7488083

[23] Harper KM , Harp SJ , Moy SS . Prenatal stress unmasks behavioral phenotypes in genetic mouse models of neurodevelopmental disorders. Front Behav Neurosci. 2023;17:1271225. 10.3389/fnbeh.2023.1271225 37809038 PMC10556231

[24] Ijomone OM , Weishaupt A‐K , Michaelis V , Ijomone OK , Bornhorst J . p38‐ and ERK‐MAPK signalling modulate developmental neurotoxicity of nickel and vanadium in the *Caenorhabditis elegans* model. Kinases and Phosphatases. 2024;2(1):28‐42. 10.3390/kinasesphosphatases2010003

[25] Ijomone OM , Ifenatuoha CW , Aluko OM , Ijomone OK , Aschner M . The aging brain: impact of heavy metal neurotoxicity. Crit Rev Toxicol. 2020;50(9):801‐814. 10.1080/10408444.2020.1838441 33210961

[26] Ijomone OM , Olung NF , Akingbade GT , Okoh COA , Aschner M . Environmental influence on neurodevelopmental disorders: Potential association of heavy metal exposure and autism. J Trace Elem Med Biol. 2020;62:126638. 10.1016/j.jtemb.2020.126638 32891009 PMC7655547

[27] Grandjean P , Landrigan P . Developmental neurotoxicity of industrial chemicals. Lancet. 2006;368(9553):2167‐2178. 10.1016/S0140-6736(06)69665-7 17174709

[28] Ruszkiewicz JA , Pinkas A , Miah MR , et al. *C. elegans* as a model in developmental neurotoxicology. Toxicol Appl Pharmacol. 2018;354:126‐135. 10.1016/j.taap.2018.03.016 29550512 PMC6087488

[29] Skogheim TS , Weyde KVF , Engel SM , et al. Metal and essential element concentrations during pregnancy and associations with autism spectrum disorder and attention‐deficit/hyperactivity disorder in children. Environ Int. 2021;152:106468. 10.1016/j.envint.2021.106468 33765546

[30] Yasuda H , Tsutsui T . Metallomics analysis for early assessment and individualized intervention of neurodevelopmental disorders. Metallomics. 2022;14(9):mfac067. 10.1093/mtomcs/mfac067 36087072

[31] Breysse PN *ATSDR 2020: Toxicological Profile for Lead*. Regulations. Published Dec 23, 2024. https://www.regulations.gov/document/EPA-HQ-OPPT-2024-0085-0176

[32] ATSDR AfTSaDR . Toxicological Profile for Mercury. ATSDR; Published Nov 05 2024. https://wwwn.cdc.gov/TSP/ToxProfiles/ToxProfiles.aspx?id=115&tid=24

[33] Ijomone OK , Ukwubile II , Aneke VO , et al. Glial perturbation in metal neurotoxicity: implications for brain disorders. Neuroglia. 2025;6(1):4. 10.3390/neuroglia6010004

[34] Nguyen T . Total number of synapses in the adult human neocortex. Undergraduate J Math Model: One+Two. 2013;3(1):26. 10.5038/2326-3652.3.1.26

[35] Piechotta K , Dudanova I , Missler M . The resilient synapse: insights from genetic interference of synaptic cell adhesion molecules. Cell Tissue Res. 2006;326(2):617‐642. 10.1007/s00441-006-0267-4 16855838

[36] Akins MR , Biederer T . Cell‐cell interactions in synaptogenesis. Curr Opin Neurobiol. 2006;16(1):83‐89. 10.1016/j.conb.2006.01.009 16427268

[37] Olsen O , Moore KA , Fukata M , et al. Neurotransmitter release regulated by a MALS–liprin‐α presynaptic complex. J Cell Biol. 2005;170(7):1127‐1134. 10.1083/jcb.200503011 16186258 PMC2171538

[38] Zhang H , Lei M , Zhang Y , et al. Phosphorylation of Doc2 by EphB2 modulates Munc13‐mediated SNARE complex assembly and neurotransmitter release. Sci Adv. 2024;10(20):eadi7024. 10.1126/sciadv.adi7024 38758791 PMC11100570

[39] Südhof TC . The cell biology of synapse formation. J Cell Biol. 2021;220(7):e202103052. 10.1083/jcb.202103052 34086051 PMC8186004

[40] Liu J , Misra A , Reddy MVVVS , White MA , Ren G , Rudenko G . Structural plasticity of neurexin 1α: implications for its role as synaptic organizer. J Mol Biol. 2018;430(21):4325‐4343. 30193986 10.1016/j.jmb.2018.08.026 PMC6223652

[41] McMahon SA , Díaz E . Mechanisms of excitatory synapse maturation by trans‐synaptic organizing complexes. Curr Opin Neurobiol. 2011;21(2):221‐227. 10.1016/j.conb.2010.12.005 21242087 PMC3085653

[42] de Wit J , Ghosh A . Specification of synaptic connectivity by cell surface interactions. Nat Rev Neurosci. 2016;17(1):4. 10.1038/nrn.2015.3 26656254

[43] Jang S , Lee H , Kim E . Synaptic adhesion molecules and excitatory synaptic transmission. Curr Opin Neurobiol. 2017;45:45‐50. 10.1016/j.conb.2017.03.005 28390263

[44] Ramirez JJ , Bindu DS , Eroglu C . Building and destroying synaptic bridges: how do Hevin/Sparcl1, SPARC, and MDGAs modify trans‐synaptic neurexin‐neuroligin interactions? Structure. 2021;29(7):635‐637. 10.1016/j.str.2021.06.011 34214438

[45] Gan KJ , Südhof TC . SPARCL1 promotes excitatory but not inhibitory synapse formation and function independent of neurexins and neuroligins. J Neurosci. 2020;40(42):8088‐8102. 10.1523/JNEUROSCI.0454-20.2020 32973045 PMC7574652

[46] Chanda S , Hale WD , Zhang B , Wernig M , Südhof TC . Unique versus redundant functions of neuroligin genes in shaping excitatory and inhibitory synapse properties. J Neurosci. 2017;37(29):6816‐6836. 10.1523/JNEUROSCI.0125-17.2017 28607166 PMC5518416

[47] Varoqueaux F , Aramuni G , Rawson RL , et al. Neuroligins determine synapse maturation and function. Neuron. 2006;51(6):741‐754. 10.1016/j.neuron.2006.09.003 16982420

[48] Lardi‐Studler B , Fritschy JM . Matching of pre‐ and postsynaptic specializations during synaptogenesis. Neuroscientist. 2007;13(2):115‐126. 10.1177/1073858406296803 17404372

[49] Bickford ME . Synaptic organization of the dorsal lateral geniculate nucleus. Eur J Neurosci. 2019;49(7):938‐947. 10.1111/ejn.13917 29575193 PMC6157005

[50] Shepherd GM . Symposium overview and historical perspective: dendrodendritic synapses: past, present, and future. Ann NY Acad Sci. 2009;1170:215‐223. 10.1111/j.1749-6632.2009.03937.x 19686140 PMC3819211

[51] Südhof TC . Synaptic neurexin complexes: a molecular code for the logic of neural circuits. Cell. 2017;171(4):745‐769. 10.1016/j.cell.2017.10.024 29100073 PMC5694349

[52] Harris KM , Weinberg RJ . Ultrastructure of synapses in the mammalian brain. Cold Spring Harbor Perspect Biol. 2012;4(5):a005587. 10.1101/cshperspect.a005587 PMC3331701 22357909

[53] Zuber B , Nikonenko I , Klauser P , Muller D , Dubochet J . The mammalian central nervous synaptic cleft contains a high density of periodically organized complexes. Pro Natl Acad Sci. 2005;102(52):19192‐19197. 10.1073/pnas.0509527102 PMC1323199 16354833

[54] Nguyen T , Südhof TC . Binding properties of neuroligin 1 and neurexin 1β reveal function as heterophilic cell adhesion molecules. J Biol Chem. 1997;272(41):26032‐26039. 10.1074/jbc.272.41.26032 9325340

[55] Ullrich B , Ushkaryov YA , Südhof TC . Cartography of neurexins: more than 1000 isoforms generated by alternative splicing and expressed in distinct subsets of neurons. Neuron. 1995;14(3):497‐507. 10.1016/0896-6273(95)90306-2 7695896

[56] Taniguchi H , Gollan L , Scholl FG , et al. Silencing of neuroligin function by postsynaptic neurexins. J Neurosci. 2007;27(11):2815‐2824. 10.1523/JNEUROSCI.0032-07.2007 17360903 PMC2839889

[57] Berninghausen O , Rahman MA , Silva JP , Davletov B , Hopkins C , Ushkaryov YA . Neurexin Iβ and neuroligin are localized on opposite membranes in mature central synapses. J Neurochem. 2007;103(5):1855‐1863. 10.1111/j.1471-4159.2007.04918.x 17868325 PMC2517655

[58] Bottos A , Rissone A , Bussolino F , Arese M . Neurexins and neuroligins: synapses look out of the nervous system. Cell Mol Life Sci. 2011;68(16):2655‐2666. 10.1007/s00018-011-0664-z 21394644 PMC11115133

[59] Shan D , Song Y , Zhang Y , et al. Neurexin dysfunction in neurodevelopmental and neuropsychiatric disorders: a PRIMSA‐based systematic review through iPSC and animal models. Front Behav Neurosci. 2024;18:1297374. 10.3389/fnbeh.2024.1297374 38380150 PMC10876810

[60] Ichtchenko K , Hata Y , Nguyen T , et al. Neuroligin 1: A splice site‐specific ligand for β‐neurexins. Cell. 1995;81(3):435‐443. 10.1016/0092-8674(95)90396-8 7736595

[61] Wang J , Sudhof T , Wernig M . Distinct mechanisms control the specific synaptic functions of neuroligin 1 and neuroligin 2. EMBO Rep. 2025;26(3):860‐879. 10.1038/s44319-024-00286-4 39747663 PMC11811269

[62] Lisé MF , El‐Husseini A . The neuroligin and neurexin families: from structure to function at the synapse. Cell Mol Life Sci. 2006;63:1833‐1849. 10.1007/s00018-006-6061-3 16794786 PMC11136152

[63] Maxeiner S , Benseler F , Brose N , Krasteva‐Christ G . Of humans and gerbils—independent diversification of neuroligin‐4 into X‐and Y‐Specific genes in primates and rodents. Front Mol Neurosci. 2022;15:838262. 10.3389/fnmol.2022.838262 35431802 PMC9005811

[64] Born G , Grayton HM , Langhorst H , et al. Genetic targeting of NRXN2 in mice unveils role in excitatory cortical synapse function and social behaviors. Front Synaptic Neurosci. 2015;7:3. 10.3389/fnsyn.2015.00003 25745399 PMC4333794

[65] Zhang B , Seigneur E , Wei P , Gokce O , Morgan J , Südhof TC . Developmental plasticity shapes synaptic phenotypes of autism‐associated neuroligin‐3 mutations in the calyx of Held. Mol Psychiatry. 2017;22(10):1483‐1491. 10.1038/mp.2016.157 27725662 PMC5687809

[66] Boucard AA , Chubykin AA , Comoletti D , Taylor P , Südhof TC . A splice code for trans‐synaptic cell adhesion mediated by binding of neuroligin 1 to α‐ and β‐neurexins. Neuron. 2005;48(2):229‐236. 10.1016/j.neuron.2005.08.026 16242404

[67] Reissner C , Klose M , Fairless R , Missler M . Mutational analysis of the neurexin/neuroligin complex reveals essential and regulatory components. Proc Natl Acad Sci. 2008;105(39):15124‐15129. 10.1073/pnas.0801639105 18812509 PMC2551626

[68] Araç D , Boucard AA , Özkan E , et al. Structures of neuroligin‐1 and the neuroligin‐1/neurexin‐1β complex reveal specific protein‐protein and protein‐Ca2+ interactions. Neuron. 2007;56(6):992‐1003. 10.1016/j.neuron.2007.12.002 18093522

[69] Graf ER , Kang Y , Hauner AM , Craig AM . Structure function and splice site analysis of the synaptogenic activity of the neurexin‐1β LNS domain. J Neurosci. 2006;26(16):4256‐4265. 10.1523/JNEUROSCI.1253-05.2006 16624946 PMC2826202

[70] Iijima T , Wu K , Witte H , et al. SAM68 regulates neuronal activity‐dependent alternative splicing of neurexin‐1. Cell. 2011;147(7):1601‐1614. 10.1016/j.cell.2011.11.028 22196734 PMC3246220

[71] Lalo U , Koh W , Lee CJ , Pankratov Y . The tripartite glutamatergic synapse. Neuropharmacology. 2021;199:108758. 10.1016/j.neuropharm.2021.108758 34433089

[72] Heller JP , Rusakov DA . The nanoworld of the tripartite synapse: insights from super‐resolution microscopy. Front Cell Neurosci. 2017;11:374. 10.3389/fncel.2017.00374 29225567 PMC5705901

[73] Chelini G , Pantazopoulos H , Durning P , Berretta S . The tetrapartite synapse: a key concept in the pathophysiology of schizophrenia. Eur Psychiatry. 2018;50:60‐69. 10.1016/j.eurpsy.2018.02.003 29503098 PMC5963512

[74] Stasenko SV , Kazantsev VB . Spiking neural network with tetrapartite synapse. Springer. 2023;1120:83‐92. 10.1007/978-3-031-44865-2_9

[75] Singh SK , Stogsdill JA , Pulimood NS , et al. Astrocytes assemble thalamocortical synapses by bridging NRX1α and NL1 via hevin. Cell. 2016;164(1‐2):183‐196. 10.1016/j.cell.2015.11.034 26771491 PMC4715262

[76] Carstens KE , Phillips ML , Pozzo‐Miller L , Weinberg RJ , Dudek SM . Perineuronal nets suppress plasticity of excitatory synapses on CA2 pyramidal neurons. J Neurosci. 2016;36(23):6312‐6320. 10.1523/JNEUROSCI.0245-16.2016 27277807 PMC4899529

[77] Hug L , Mpai R . Optimization of experimental conditions to characterize perineuronal nets in the human cerebellum. McGill Science Undergraduate Res J. 2024;19(1):1‐5. 10.26443/msurj.v19i1.224

[78] Cao X , Tabuchi K . Functions of synapse adhesion molecules neurexin/neuroligins and neurodevelopmental disorders. Neurosci Res. 2017;116:3‐9. 10.1016/j.neures.2016.09.005 27664583

[79] Ribeiro LF , Verpoort B , de Wit J . Trafficking mechanisms of synaptogenic cell adhesion molecules. Mol Cell Neurosci. 2018;91:34‐47. 10.1016/j.mcn.2018.04.003 29631018

[80] Zhang JS , Honkaniemi J , Yang T , Yeo TT , Longo FM . LAR tyrosine phosphatase receptor: a developmental isoform is present in neurites and growth cones and its expression is regional‐and cell‐specific. Mol Cell Neurosci. 1998;10(5‐6):271‐286. 10.1006/mcne.1998.0663 9604206

[81] Boxer EE , Aoto J . Neurexins and their ligands at inhibitory synapses. Front Synaptic Neurosci. 2022;14:1087238. 10.3389/fnsyn.2022.1087238 36618530 PMC9812575

[82] Schreiner D , Scheiffele P . Neuroligins and neurexins. In: Rubenstein J , Rakic P , Chen B , Kwan KY , Cline HT , Cardin J , eds., Synapse Development and Maturation. Elsevier; 2020:193‐212 10.1016/B978-0-12-823672-7.00008-9

[83] Hirano S , Takeichi M . Cadherins in brain morphogenesis and wiring. Physiol Rev. 2012;92(2):597‐634. 10.1152/physrev.00014.2011 22535893

[84] Nguyen L , Hippenmeyer S . Cellular and molecular control of neuronal migration. Springer; 2014.

[85] Takahashi H , Craig AM . Protein tyrosine phosphatases PTPδ, PTPσ, and LAR: presynaptic hubs for synapse organization. Trends Neurosci. 2013;36(9):522‐534. 10.1016/j.tins.2013.06.002 23835198 PMC3789601

[86] Michetti C , Falace A , Benfenati F , Fassio A . Synaptic genes and neurodevelopmental disorders: from molecular mechanisms to developmental strategies of behavioral testing. Neurobiol Dis. 2022;173:105856. 10.1016/j.nbd.2022.105856 36070836

[87] Blum D , Lopes LV . Stabilizing synapses. Science. 2021;374(6568):684‐685. 10.1126/science.abm3902 34735229

[88] Sanes JR , Lichtman JW . Development of the vertebrate neuromuscular junction. Annu Rev Neurosci. 1999;22(1):389‐442. 10.1146/annurev.neuro.22.1.389 10202544

[89] Garcia N , Lanuza MA , Tomàs M , et al. PKA and PKC balance in synapse elimination during neuromuscular junction development. Cells. 2021;10(6):1384. 10.3390/cells10061384 34199823 PMC8230189

[90] Chen C , Regehr WG . Developmental remodeling of the retinogeniculate synapse. Neuron. 2000;28(3):955‐966. 10.1016/s0896-6273(00)00166-5 11163279

[91] Cong Q , Soteros BM , Wollet M , Kim JH , Sia GM . The endogenous neuronal complement inhibitor SRPX2 protects against complement‐mediated synapse elimination during development. Nature Neurosci. 2020;23(9):1067‐1078. 10.1038/s41593-020-0672-0 32661396 PMC7483802

[92] Horstkorte R , Fuss B . Cell adhesion molecules. In: Brady S , Siegal G , Albers RW , Price D , eds., Basic Neurochemistry: Principles of Molecular, Cellular, and Medical Neurobiology. Elsevier; 2012:165‐179 10.1016/B978-0-12-374947-5.00009-2

[93] Taylor SC , Ferri SL , Grewal M , et al. The role of synaptic cell adhesion molecules and associated scaffolding proteins in social affiliative behaviors. Biol Psychiatry. 2020;88(6):442‐451. 10.1016/j.biopsych.2020.02.012 32305215 PMC7442706

[94] Ozkan ED , Creson TK , Kramár EA , et al. Reduced cognition in Syngap1 mutants is caused by isolated damage within developing forebrain excitatory neurons. Neuron. 2014;82(6):1317‐1333. 10.1016/j.neuron.2014.05.015 24945774 PMC4104574

[95] Gaugler T , Klei L , Sanders SJ , et al. Most genetic risk for autism resides with common variation. Nature Genet. 2014;46(8):881‐885. 10.1038/ng.3039 25038753 PMC4137411

[96] Sanders SJ , He X , Willsey AJ , et al. Insights into autism spectrum disorder genomic architecture and biology from 71 risk loci. Neuron. 2015;87(6):1215‐1233. 10.1016/j.neuron.2015.09.016 26402605 PMC4624267

[97] Hu Z , Yang Y , Zhao Y , et al. APOE hypermethylation is associated with autism spectrum disorder in a Chinese population. Exp Ther Med. 2018;15(6):4749‐4754. 10.3892/etm.2018.6069 29844799 PMC5958870

[98] Noborn F , Sterky FH . Role of neurexin heparan sulfate in the molecular assembly of synapses – expanding the neurexin code? FEBS J. 2023;290(2):252‐265. 10.1111/febs.16251 34699130

[99] Philbrook A , Ramachandran S , Lambert CM , et al. Neurexin directs partner‐specific synaptic connectivity in *C. elegans* . eLife. 2018;7:e35692. 10.7554/eLife.35692 30039797 PMC6057746

[100] Hart MP . Stress‐Induced neuron remodeling reveals differential interplay between neurexin and environmental factors in *Caenorhabditis elegans* . Genetics. 2019;213(4):1415‐1430. 10.1534/genetics.119.302415 31558583 PMC6893388

[101] Jiang X , Sando R , Südhof TC . Multiple signaling pathways are essential for synapse formation induced by synaptic adhesion molecules. Proc Natl Acad Sci. 2021;118(3):e2000173118. 10.1073/pnas.2000173118 33431662 PMC7826368

[102] Fuccillo MV , Földy C , Gökce Ö , et al. Single‐cell mRNA profiling reveals cell‐type‐specific expression of neurexin isoforms. Neuron. 2015;87(2):326‐340. 10.1016/j.neuron.2015.06.028 26182417 PMC4733560

[103] Nguyen TM , Schreiner D , Xiao L , Traunmüller L , Bornmann C , Scheiffele P . An alternative splicing switch shapes neurexin repertoires in principal neurons versus interneurons in the mouse hippocampus. eLife. 2016;5:e22757. 10.7554/eLife.22757 27960072 PMC5213383

[104] Rudenko G . Neurexins—Versatile molecular platforms in the synaptic cleft. Curr Opin Struct Biol. 2019;54:112‐121. 10.1016/j.sbi.2019.01.009 30831539 PMC6592730

[105] Liakath‐Ali K , Südhof TC . The perils of navigating activity‐dependent alternative splicing of neurexins. Front Mol Neurosci. 2021;14:659681. 10.3389/fnmol.2021.659681 33767611 PMC7985251

[106] Han KA , Kim YJ , Yoon TH , et al. LAR‐RPTPs directly interact with neurexins to coordinate bidirectional assembly of molecular machineries. J Neurosci. 2020;40(44):8438‐8462. 10.1523/JNEUROSCI.1091-20.2020 33037075 PMC7605416

[107] Roppongi RT , Dhume SH , Padmanabhan N , et al. LRRTMs organize synapses through differential engagement of neurexin and PTPσ. Neuron. 2020;106(1):108‐125. e12. 31995730 10.1016/j.neuron.2020.01.003

[108] Kim JYV , Megat S , Moy JK , et al. Neuroligin 2 regulates spinal GABAergic plasticity in hyperalgesic priming, a model of the transition from acute to chronic pain. Pain. 2016;157(6):1314‐1324. 10.1097/j.pain.0000000000000513 26859820 PMC4920002

[109] Hata Y , Butz S , Sudhof T . CASK: a novel dlg/PSD95 homolog with an N‐terminal calmodulin‐dependent protein kinase domain identified by interaction with neurexins. J Neurosci. 1996;16(8):2488‐2494. 10.1523/JNEUROSCI.16-08-02488.1996 8786425 PMC6578772

[110] Irie M , Hata Y , Takeuchi M , et al. Binding of neuroligins to PSD‐95. Science. 1997;277(5331):1511‐1515. 10.1126/science.277.5331.1511 9278515

[111] Levy AM , Gomez‐Puertas P , Tümer Z . Neurodevelopmental disorders associated with PSD‐95 and its interaction partners. Int J Mol Sci. 2022;23(8):4390. 10.3390/ijms23084390 35457207 PMC9025546

[112] Ichtchenko K , Nguyen T , Südhof TC . Structures, alternative splicing, and neurexin binding of multiple neuroligins. J Biol Chem. 1996;271(5):2676‐2682. 10.1074/jbc.271.5.2676 8576240

[113] Bemben MA , Shipman SL , Nicoll RA , Roche KW . The cellular and molecular landscape of neuroligins. Trends Neurosci. 2015;38(8):496‐505. 10.1016/j.tins.2015.06.004 26209464 PMC9381026

[114] Kasem E , Kurihara T , Tabuchi K . Neurexins and neuropsychiatric disorders. Neurosci Res. 2018;127:53‐60. 10.1016/j.neures.2017.10.012 29221905

[115] Nguyen TA , Lehr AW , Roche KW . Neuroligins and neurodevelopmental disorders: X‐Linked genetics. Front Synaptic Neurosci. 2020;12:33. 10.3389/fnsyn.2020.00033 32848696 PMC7431521

[116] Vieira MM , Jeong J , Roche KW . The role of NMDA receptor and neuroligin rare variants in synaptic dysfunction underlying neurodevelopmental disorders. Curr Opin Neurobiol. 2021;69:93‐104. 10.1016/j.conb.2021.03.001 33823469

[117] Wang J , Gong J , Li L , et al. Neurexin gene family variants as risk factors for autism spectrum disorder. Autism Research. 2018;11(1):37‐43. 10.1002/aur.1881 29045040

[118] Gerik‐Celebi HB , Bolat H , Unsel‐Bolat G . Rare heterozygous genetic variants of NRXN and NLGN gene families involved in synaptic function and their association with neurodevelopmental disorders. Dev Neurobiol. 2024;84(3):158‐168. 10.1002/dneu.22941 38739110

[119] Rowen L , Young J , Birditt B , et al. Analysis of the human neurexin genes: alternative splicing and the generation of protein diversity. Genomics. 2002;79(4):587‐597. 10.1006/geno.2002.6734 11944992

[120] Tromp A , Mowry B , Giacomotto J . Neurexins in autism and schizophrenia‐a review of patient mutations, mouse models and potential future directions. Mol Psychiatry. 2021;26(3):747‐760. 10.1038/s41380-020-00944-8 33191396

[121] Armstrong EC , Caruso A , Servadio M , et al. Assessing the developmental trajectory of mouse models of neurodevelopmental disorders: social and communication deficits in mice with neurexin 1α deletion. Genes Brain Behav. 2020;19(4):e12630. 10.1111/gbb.12630 31823470

[122] Grayton HM , Missler M , Collier DA , Fernandes C . Altered social behaviours in neurexin 1α knockout mice resemble core symptoms in neurodevelopmental disorders. PLoS One. 2013;8(6):e67114. 10.1371/journal.pone.0067114 23840597 PMC3696036

[123] Hodge R . Human Sex‐Specific Synaptic Adhesion Proteins NLGN4X and NLGN4Y Regulate the Dual Innervation of Dendritic Spines. Thomas Jefferson University; 2021.

[124] Craig AM , Kang Y . Neurexin‐neuroligin signaling in synapse development. Curr Opin Neurobiol. 2007;17(1):43‐52. 10.1016/j.conb.2007.01.011 17275284 PMC2820508

[125] Trobiani L , Meringolo M , Diamanti T , et al. The neuroligins and the synaptic pathway in autism spectrum disorder. Neurosci Biobehav Rev. 2020;119:37‐51. 10.1016/j.neubiorev.2020.09.017 32991906

[126] Zhao JY , Duan XL , Yang L , et al. Activity‐dependent synaptic recruitment of neuroligin 1 In spinal dorsal horn contributed to inflammatory pain. Neuroscience. 2018;388:1‐10. 10.1016/j.neuroscience.2018.06.047 30049666

[127] Ali H , Marth L , Krueger‐Burg D . Neuroligin‐2 as a central organizer of inhibitory synapses in health and disease. Sci Signaling. 2020;13(663):eabd8379. 10.1126/scisignal.abd8379 33443230

[128] Budreck EC , Scheiffele P . Neuroligin‐3 is a neuronal adhesion protein at GABAergic and glutamatergic synapses. Eur J Neurosci. 2007;26(7):1738‐1748. 10.1111/j.1460-9568.2007.05842.x 17897391

[129] Bemben MA , Nguyen QA , Wang T , Li Y , Nicoll RA , Roche KW . Autism‐associated mutation inhibits protein kinase C‐mediated neuroligin‐4X enhancement of excitatory synapses. Proc Natl Acad Sci. 2015;112(8):2551‐2556. 10.1073/pnas.1500501112 25675530 PMC4345621

[130] Nakanishi M , Nomura J , Ji X , et al. Functional significance of rare neuroligin 1 variants found in autism. PLoS Genet. 2017;13(8):e1006940. 10.1371/journal.pgen.1006940 28841651 PMC5571902

[131] Redin C , Gérard B , Lauer J , et al. Efficient strategy for the molecular diagnosis of intellectual disability using targeted high‐throughput sequencing. J Med Genet. 2014;51(11):724‐736. 10.1136/jmedgenet-2014-102554 25167861 PMC4215287

[132] Geisheker MR , Heymann G , Wang T , et al. Hotspots of missense mutation identify neurodevelopmental disorder genes and functional domains. Nature Neurosci. 2017;20(8):1043‐1051. 10.1038/nn.4589 28628100 PMC5539915

[133] Quartier A , Courraud J , Thi Ha T , et al. Novel mutations in NLGN3 causing autism spectrum disorder and cognitive impairment. Hum Mutat. 2019;40(11):2021‐2032. 10.1002/humu.23836 31184401

[134] Parente DJ , Garriga C , Baskin B , et al. Neuroligin 2 nonsense variant associated with anxiety, autism, intellectual disability, hyperphagia, and obesity. Am J Med Genet, Part A. 2017;173(1):213‐216. 10.1002/ajmg.a.37977 27865048

[135] De Jaco A , Lin MZ , Dubi N , et al. Neuroligin trafficking deficiencies arising from mutations in the α/β‐hydrolase fold protein family. J Biol Chem. 2010;285(37):28674‐28682. 10.1074/jbc.M110.139519 20615874 PMC2937894

[136] Marro SG , Chanda S , Yang N , et al. Neuroligin‐4 regulates excitatory synaptic transmission in human neurons. Neuron. 2019;103(4):617‐626.e6. 10.1016/j.neuron.2019.05.043. 31257103 PMC6706319

[137] Nguyen TA , Wu K , Pandey S , et al. A cluster of autism‐associated variants on X‐linked NLGN4X functionally resemble NLGN4Y. Neuron. 2020;106(5):759‐768.e7. 10.1016/j.neuron.2020.03.008. 32243781 PMC7491604

[138] Gilman SR , Iossifov I , Levy D , Ronemus M , Wigler M , Vitkup D . Rare de novo variants associated with autism implicate a large functional network of genes involved in formation and function of synapses. Neuron. 2011;70(5):898‐907. 10.1016/j.neuron.2011.05.021 21658583 PMC3607702

[139] Sanders SJ , Ercan‐Sencicek AG , Hus V , et al. Multiple recurrent de novo CNVs, including duplications of the 7q11.23 Williams syndrome region, are strongly associated with autism. Neuron. Jun 9 2011;70(5):863‐885. 10.1016/j.neuron.2011.05.002 21658581 PMC3939065

[140] Jamain S , Quach H , Betancur C , et al. Mutations of the X‐linked genes encoding neuroligins NLGN3 and NLGN4 are associated with autism. Nature Genet. 2003;34(1):27‐29. 10.1038/ng1136 12669065 PMC1925054

[141] Kopp N , Amarillo I , Martinez‐Agosto J , Quintero‐Rivera F . Pathogenic paternally inherited NLGN4X deletion in a female with autism spectrum disorder: Clinical, cytogenetic, and molecular characterization. Am J Med Genet, Part A. 2021;185(3):894‐900. 10.1002/ajmg.a.62025 33369065

[142] Doherty P , Williams E , Walsh FS . A soluble chimeric form of the L1 glycoprotein stimulates neurite outgrowth. Neuron. 1995;14(1):57‐66. 10.1016/0896-6273(95)90240-6 7826641

[143] Yamagata M , Duan X , Sanes JR . Cadherins interact with synaptic organizers to promote synaptic differentiation. Front Mol Neurosci. 2018;11:142. 10.3389/fnmol.2018.00142 29760652 PMC5936767

[144] Rivero O , Selten MM , Sich S , et al. Cadherin‐13, a risk gene for ADHD and comorbid disorders, impacts GABAergic function in hippocampus and cognition. Transl Psychiatry. 2015;5(10):e655. 10.1038/tp.2015.152 26460479 PMC4930129

[145] Tamura K , Shan WS , Hendrickson WA , Colman DR , Shapiro L . Structure‐function analysis of cell adhesion by neural (N‐) cadherin. Neuron. 1998;20(6):1153‐1163. 10.1016/s0896-6273(00)80496-1 9655503

[146] Benson D . Making memories stick: cell‐adhesion molecules in synaptic plasticity. Trends Cell Biol. 2000;10(11):473‐482. 10.1016/s0962-8924(00)01838-9 11050419

[147] Pettitt J . The cadherin superfamily. In: WormBoo: The Online Review of C. Elegans Biologyk. 2005:1‐9. 10.1895/wormbook.1.50.1 PMC4781240 18050421

[148] Basu R , Taylor MR , Williams ME . The classic cadherins in synaptic specificity. Cell Adh Migr. 2015;9(3):193‐201. 10.1080/19336918.2014.1000072 25837840 PMC4594527

[149] Accogli A , Calabretta S , St‐Onge J , et al. De novo pathogenic variants in N‐cadherin cause a syndromic neurodevelopmental disorder with corpus callosum, axon, cardiac, ocular, and genital defects. Am J Hum Genet. 2019;105(4):854‐868. 10.1016/j.ajhg.2019.09.005 31585109 PMC6817525

[150] Halperin D , Stavsky A , Kadir R , et al. CDH2 mutation affecting N‐cadherin function causes attention‐deficit hyperactivity disorder in humans and mice. Nat Commun. 2021;12(1):6187. 10.1038/s41467-021-26426-1 34702855 PMC8548587

[151] Liu D , Cao H , Kural KC , Fang Q , Zhang F . Integrative analysis of shared genetic pathogenesis by autism spectrum disorder and obsessive‐compulsive disorder. Biosci Rep. 2019;39(12):BSR20191942. 10.1042/BSR20191942 31808517 PMC6928520

[152] László ZI , Lele Z . Flying under the radar: CDH2 (N‐cadherin), an important hub molecule in neurodevelopmental and neurodegenerative diseases. Front Neurosci. 2022;16:972059. 10.3389/fnins.2022.972059 36213737 PMC9539934

[153] Piprek RP , Kolasa M , Podkowa D , Kloc M , Kubiak JZ . N‐cadherin is critical for the survival of germ cells, the formation of steroidogenic cells, and the architecture of developing mouse gonads. Cells. 2019;8(12):1610. 10.3390/cells8121610 31835801 PMC6952792

[154] Oliver C , González CA , Alvial G , Flores CA , Rodríguez EM , Bátiz LF . Disruption of CDH2/N‐cadherin‐based adherens junctions leads to apoptosis of ependymal cells and denudation of brain ventricular walls. J Neuropathol Exp Neurol. 2013;72(9):846‐860. 10.1097/NEN.0b013e3182a2d5fe 23965744

[155] Rebman JK , Kirchoff KE , Walsh GS . Cadherin‐2 is required cell autonomously for collective migration of facial branchiomotor neurons. PLoS One. 2016;11(10):e0164433. 10.1371/journal.pone.0164433 27716840 PMC5055392

[156] McGregor NW , Lochner C , Stein DJ , Hemmings SMJ . Polymorphisms within the neuronal cadherin (CDH2) gene are associated with obsessive‐compulsive disorder (OCD) in a South African cohort. Metab Brain Dis. 2016;31:191‐196. 10.1007/s11011-015-9693-x 26093892

[157] Smith KR , Jones KA , Kopeikina KJ , et al. Cadherin‐10 maintains excitatory/inhibitory ratio through interactions with synaptic proteins. J Neurosci. 2017;37(46):11127‐11139. 10.1523/JNEUROSCI.1153-17.2017 29030434 PMC5688522

[158] Williams ME , Wilke SA , Daggett A , et al. Cadherin‐9 regulates synapse‐specific differentiation in the developing hippocampus. Neuron. 2011;71(4):640‐655. 10.1016/j.neuron.2011.06.019 21867881 PMC3272880

[159] Lilja J , Ivaska J . Integrin activity in neuronal connectivity. J Cell Sci. 2018;131(12):jcs212803. 10.1242/jcs.212803 29907643

[160] Moreno‐Layseca P , Icha J , Hamidi H , Ivaska J . Integrin trafficking in cells and tissues. Nature Cell Biol. 2019;21(2):122‐132. 10.1038/s41556-018-0223-z 30602723 PMC6597357

[161] Kadry YA , Calderwood DA . Chapter 22: structural and signaling functions of integrins. Biochim Biophys Acta (BBA) ‐ Biomembranes. 2020;1862(5):183206. 10.1016/j.bbamem.2020.183206 31991120 PMC7063833

[162] Dohn MR , Kooker CG , Bastarache L , et al. The gain‐of‐function integrin β3 Pro33 variant alters the serotonin system in the mouse brain. J Neurosci. 2017;37(46):11271‐11284. 10.1523/JNEUROSCI.1482-17.2017 29038237 PMC5688530

[163] Yamaguchi Y , Pasquale EB . Eph receptors in the adult brain. Curr Opin Neurobiol. 2004;14(3):288‐296. 10.1016/j.conb.2004.04.003 15194108

[164] Aoto J , Chen L . Bidirectional ephrin/Eph signaling in synaptic functions. Brain Res. 2007;1184:72‐80. 10.1016/j.brainres.2006.11.033 17166489 PMC2170431

[165] Klein R . Bidirectional modulation of synaptic functions by Eph/ephrin signaling. Nature Neurosci. 2009;12(1):15‐20. 10.1038/nn.2231 19029886

[166] Cramer KS , Miko IJ . Eph‐ephrin signaling in nervous system development. F1000Research. 2016;5:413. 10.12688/f1000research.7417.1 PMC4821289 27092247

[167] Kania A , Klein R . Mechanisms of ephrin‐Eph signalling in development, physiology and disease. Nat Rev Mol Cell Biol. 2016;17(4):240‐256. 10.1038/nrm.2015.16 26790531

[168] Sheleg M , Yochum CL , Wagner GC , Zhou R , Richardson JR . Ephrin‐A5 deficiency alters sensorimotor and monoaminergic development. Behav Brain Res. 2013;236(1):139‐147. 10.1016/j.bbr.2012.08.032 22954718 PMC3482281

[169] Wurzman R , Forcelli PA , Griffey CJ , Kromer LF . Repetitive grooming and sensorimotor abnormalities in an ephrin‐A knockout model for autism spectrum disorders. Behav Brain Res. 2015;278:115‐128. 10.1016/j.bbr.2014.09.012 25281279 PMC4382445

[170] Arnall S , Cheam LY , Smart C , et al. Abnormal strategies during visual discrimination reversal learning in ephrin‐A2(‐/‐) mice. Behav Brain Res. 2010;209(1):109‐113. 10.1016/j.bbr.2010.01.023 20100519

[171] Rutz HLH , Rothblat LA . Intact and impaired executive abilities in the BTBR mouse model of autism. Behav Brain Res. 2012;234(1):33‐37. 10.1016/j.bbr.2012.05.048 22677272

[172] Jomova K , Makova M , Alomar SY , et al. Essential metals in health and disease. Chemico‐Biol Interact. 2022;367:110173. 10.1016/j.cbi.2022.110173 36152810

[173] Bradbury MWB . Chapter 20: An approach to study of transport of trace metals at the blood‐brain barrier. In: Armin E , Rainer L , Hans‐Joachim R , eds., Circumventricular Organs and Brain Fluid Environment. Elsevier; 1992:133‐138vol. Progress in Brain Research. 10.1016/s0079-6123(08)62327-4 1410398

[174] Pellowski D , Ebert F , Bornhorst J , Schwerdtle T . Zinc‐modulated bidirectional copper transfer across the blood‐brain barrier in a porcine brain capillary endothelial cell culture model system. J Trace Elem Med Biol. 2024;86:127547. 10.1016/j.jtemb.2024.127547 39442467

[175] Martinez‐Finley EJ , Chakraborty S , Fretham SJB , Aschner M . Cellular transport and homeostasis of essential and nonessential metals. Metallomics. 2012;4(7):593‐605. 10.1039/c2mt00185c 22337135 PMC4936191

[176] Lucchini RG , Aschner M , Bellinger DC , Caito SW . Neurotoxicology of metals. In: Nordberg GunnarF , Bruce A , eds., Handbook on the Toxicology of Metals. Elsevier; 2015:299‐311 10.1016/B978-0-444-59453-2.00015-9

[177] Zahoor SM , Ishaq S , Ahmed T . Neurotoxic effects of metals on blood brain barrier impairment and possible therapeutic approaches. Vitam Horm. 2024;126:1‐24. 10.1016/bs.vh.2024.04.003 39029969

[178] Tjälve H , Henriksson J . Uptake of metals in the brain via olfactory pathways. Neurotoxicology. 1999;20(2‐3):181‐195. 10385882

[179] Tjälve H , Tallkvist J . The olfactory pathway as a route of entry of metals into the brain. Metal Ions and Neurodegenerative Disorders. World Scientific; 2003:67‐98. 10.1142/9789812796691_0003

[180] Chalansonnet M , Carabin N , Boucard S , et al. Study of potential transfer of aluminum to the brain via the olfactory pathway. Toxicol Lett. 2018;283:77‐85. 10.1016/j.toxlet.2017.11.027 29180288

[181] Althomali RH , Abbood MA , Saleh EAM , et al. Exposure to heavy metals and neurocognitive function in adults: a systematic review. Environ Sci Eur. 2024;36(1):18. 10.1186/s12302-024-00843-7

[182] Neal AP , Guilarte TR . Mechanisms of lead and manganese neurotoxicity. Toxicol Res. 2013;2(2):99‐114. 10.1039/C2TX20064C PMC4338437 25722848

[183] Verkhratsky A , Nedergaard M . Physiology of astroglia. Physiol Rev. 2018;98(1):239‐389. 10.1152/physrev.00042.2016 29351512 PMC6050349

[184] Valles SL , Singh SK , Campos‐Campos J , et al. Functions of astrocytes under normal conditions and after a brain disease. Int J Mol Sci. 2023;24(9):8434. 10.3390/ijms24098434 37176144 PMC10179527

[185] Verkhratsky A , Rose CR . Na(+)‐dependent transporters: the backbone of astroglial homeostatic function. Cell Calcium. 2020;85:102136. 10.1016/j.ceca.2019.102136 31835178

[186] Li B , Xia M , Zorec R , Parpura V , Verkhratsky A . Astrocytes in heavy metal neurotoxicity and neurodegeneration. Brain Res. 2021;1752:147234. 10.1016/j.brainres.2020.147234 33412145 PMC8999909

[187] Milatovic D , Yin Z , Gupta RC , et al. Manganese induces oxidative impairment in cultured rat astrocytes. Toxicol Sci. 2007;98(1):198‐205. 10.1093/toxsci/kfm095 17468184

[188] Yubolphan R , Phuagkhaopong S , Sangpairoj K , Sibmooh N , Power C , Vivithanaporn P . Intracellular nickel accumulation induces apoptosis and cell cycle arrest in human astrocytic cells. Metallomics. 2020;13(1):mfaa006. 10.1093/mtomcs/mfaa006 33570137

[189] Baraibar AM , de Pascual R , Carretero VJ , Liccardi N , Juárez NH , Hernández‐Guijo JM . Aluminum alters excitability by inhibiting calcium, sodium, and potassium currents in bovine chromaffin cells. J Neurochem. 2023;165(2):162‐176. 10.1111/jnc.15784 36800503

[190] Busselberg D , Platt B , Michael D , Carpenter DO , Haas HL . Mammalian voltage‐activated calcium channel currents are blocked by Pb2+, Zn2+, and Al3+. J Neurophysiol. 1994;71(4):1491‐1497. 10.1152/jn.1994.71.4.1491 8035230

[191] Julka D , Gill KD . Altered calcium homeostasis: a possible mechanism of aluminium‐induced neurotoxicity. Biochim Biophys Acta (BBA)‐Mol Basis Dis. 1996;1315(1):47‐54. 10.1016/0925-4439(95)00100-x 8611646

[192] Carmona A , Roudeau S , Ortega R . Molecular mechanisms of environmental metal neurotoxicity: a focus on the interactions of metals with synapse structure and function. Toxics. 2021;9(9):198. 10.3390/toxics9090198 34564349 PMC8471991

[193] Sadiq S , Ghazala Z , Chowdhury A , Büsselberg D . Metal toxicity at the synapse: presynaptic, postsynaptic, and long‐term effects. J Toxicol. 2012;2012:132671. 10.1155/2012/132671 22287959 PMC3263637

[194] Minami A , Takeda A , Nishibaba D , Takefuta S , Oku N . Cadmium toxicity in synaptic neurotransmission in the brain. Brain Res. 2001;894(2):336‐339. 10.1016/s0006-8993(01)02022-4 11251212

[195] Marchetti C . Interaction of metal ions with neurotransmitter receptors and potential role in neurodiseases. BioMetals. 2014;27(6):1097‐1113. 10.1007/s10534-014-9791-y 25224737

[196] Chandravanshi LP , Agrawal P , Darwish HW , Trigun SK . Impairments of spatial memory and N‐methyl‐D‐aspartate receptors and their postsynaptic signaling molecules in the hippocampus of developing rats induced by As, Pb, and Mn mixture exposure. Brain Sci. 2023;13(12):1715. 10.3390/brainsci13121715 38137163 PMC10742016

[197] Khedr NF , Talkan OFA . New insights into arsenic, lead, and iron neurotoxicity: activation of MAPK signaling pathway and oxidative stress. J Biochem Mol Toxicol. 2022;36(6):e23040. 10.1002/jbt.23040 35307918

[198] Zhao F , Wang Z , Liao Y , Wang G , Jin Y . Alterations of NMDA and AMPA receptors and their signaling apparatus in the hippocampus of mouse offspring induced by developmental arsenite exposure. J Toxicol Sci. 2019;44(11):777‐788. 10.2131/jts.44.777 31708534

[199] Del Pino J , Zeballos G , Anadon MJ , et al. Muscarinic M1 receptor partially modulates higher sensitivity to cadmium‐induced cell death in primary basal forebrain cholinergic neurons: a cholinesterase variants dependent mechanism. Toxicology. Jun 15 2016;361‐362:1‐11. 10.1016/j.tox.2016.06.019 27377441

[200] Moyano P , de Frias M , Lobo M , et al. Cadmium induced ROS alters M1 and M3 receptors, leading to SN56 cholinergic neuronal loss, through AChE variants disruption. Toxicology. 2018;394:54‐62. 10.1016/j.tox.2017.12.006 29253600

[201] Gorini F , Muratori F , Morales MA . The role of heavy metal pollution in neurobehavioral disorders: a focus on autism. Review Journal of Autism and Developmental Disorders. 2014;1:354‐372. 10.1007/s40489-014-0028-3

[202] Frye RE , Cakir J , Rose S , et al. Early life metal exposure dysregulates cellular bioenergetics in children with regressive autism spectrum disorder. Transl Psychiatry. 2020;10(1):223. 10.1038/s41398-020-00905-3 32636364 PMC7341836

[203] Chou C‐H , Harper C Toxicological profile for arsenic. CDC STACK. Published 08/01/2007. https://stacks.cdc.gov/view/cdc/11481

[204] Ashizawa A , Faroon O , Ingerman L , Jenkins K , Tucker P , Wright S . Toxicological Profile for Cadmium. Agency for Toxic Substances and Disease Registry (US); 2012. 24049863

[205] Al‐Farsi YM , Waly MI , Al‐Sharbati MM , et al. Levels of heavy metals and essential minerals in hair samples of children with autism in Oman: a case‐control study. Biol Trace Elem Res. 2013;151(2):181‐186. 10.1007/s12011-012-9553-z 23188679

[206] Nayak S , Sahu S , John J , Patra S . Hair and urine lead, cadmium, nickel, and arsenic levels in children with attention‐deficit hyperactivity disorder: A case–control study in a tertiary care hospital in eastern India. Precision Med Sci. 2023;12(3):153‐158. 10.1002/prm2.12103

[207] Beaudin SA , Strupp BJ , Strawderman M , Smith DR . Early postnatal manganese exposure causes lasting impairment of selective and focused attention and arousal regulation in adult rats. Environ Health Perspect. 2017;125(2):230‐237. 10.1289/EHP258 27384154 PMC5289906

[208] Schneider JS , Decamp E , Clark K , Bouquio C , Syversen T , Guilarte TR . Effects of chronic manganese exposure on working memory in non‐human primates. Brain Res. 2009;1258:86‐95. 10.1016/j.brainres.2008.12.035 19133246 PMC2659542

[209] Ouisselsat M , Maidoumi S , Elmaouaki A , Lekouch N , Pineau A , Sedki A . Hair trace elements and mineral content in Moroccan children with autism spectrum disorder: a case‐control study. Biol Trace Elem Res. 2023;201(6):2701‐2710. 10.1007/s12011-022-03365-6 35896886

[210] Ma J , Wu J , Li H , Wang J , Han J , Zhang R . Association between essential metal elements and the risk of autism in Chinese Han population. Biol Trace Elem Res. 2022;200(2):505‐515. 10.1007/s12011-021-02690-6 33797704

[211] Pavăl D . A dopamine hypothesis of autism spectrum disorder. Dev Neurosci. 2017;39(5):355‐360. 10.1159/000478725 28750400

[212] Ijomone OM , Aluko OM , Okoh COA , Martins Jr. AC , Aschner M . Role for calcium signaling in manganese neurotoxicity. J Trace Elem Med Biol. 2019;56:146‐155. 10.1016/j.jtemb.2019.08.006 31470248

[213] Ijomone OM , Miah MR , Peres TV , Nwoha PU , Aschner M . Null allele mutants of trt‐1, the catalytic subunit of telomerase in *Caenorhabditis elegans*, are less sensitive to Mn‐induced toxicity and DAergic degeneration. Neurotoxicology. 2016;57:54‐60. 10.1016/j.neuro.2016.08.016 27593554

[214] Peres TV , Schettinger MRC , Chen P , et al. Manganese‐induced neurotoxicity: a review of its behavioral consequences and neuroprotective strategies. BMC Pharmacol Toxicol. 2016;17(1):57. 10.1186/s40360-016-0099-0 27814772 PMC5097420

[215] Torti SV , Torti FM . Iron: the cancer connection. Mol Aspects Med. 2020;75:100860. 10.1016/j.mam.2020.100860 32340745 PMC9107937

[216] Georgieff MK . Iron deficiency in pregnancy. Am J Obstet Gynecol. 2020;223(4):516‐524. 10.1016/j.ajog.2020.03.006 32184147 PMC7492370

[217] Georgieff MK . The role of iron in neurodevelopment: fetal iron deficiency and the developing hippocampus. Biochem Soc Trans. 2008;36(Pt 6):1267‐1271. 10.1042/BST0361267 19021538 PMC2711433

[218] Chen L , Guo X , Hou C , et al. The causal association between iron status and the risk of autism: a Mendelian randomization study. Front Nutr. 2022;9:957600. 10.3389/fnut.2022.957600 36407516 PMC9669792

[219] Latif A , Heinz P , Cook R . Iron deficiency in autism and Asperger syndrome. Autism. 2002;6:103‐114. 10.1177/1362361302006001008 11918106

[220] Hare DJ , Arora M , Jenkins NL , Finkelstein DI , Doble PA , Bush AI . Is early‐life iron exposure critical in neurodegeneration? Nat Rev Neurol. 2015;11:536‐544. 10.1038/nrneurol.2015.100 26100754

[221] Lepeta K , Lourenco MV , Schweitzer BC , et al. Synaptopathies: synaptic dysfunction in neurological disorders ‐ a review from students to students. J Neurochem. 2016;138(6):785‐805. 10.1111/jnc.13713 27333343 PMC5095804

[222] Südhof TC , Malenka RC . Understanding synapses: past, present, and future. Neuron. 2008;60(3):469‐476. 10.1016/j.neuron.2008.10.011 18995821 PMC3243741

[223] LeDoux J . Synaptic Self: How Our Brains Become Who We Are. Penguin; 2003.

[224] Garí M , Grzesiak M , Krekora M , et al. Prenatal exposure to neurotoxic metals and micronutrients and neurodevelopmental outcomes in early school age children from Poland. Environ Res. 2022;204(Pt B):112049. 10.1016/j.envres.2021.112049 34520749

[225] Liu CH , Huang CY , Huang CC . Occupational neurotoxic diseases in Taiwan. Saf Health Work. 2012;3(4):257‐267. 10.5491/SHAW.2012.3.4.257 23251841 PMC3521924

[226] Gunderson JT , Peppriell AE , Krout IN , Vorojeikina D , Rand MD . Neuroligin‐1 is a mediator of methylmercury neuromuscular toxicity. Toxicol Sci. 2021;184(2):236‐251. 10.1093/toxsci/kfab114 34546366 PMC8633903

[227] Hunter JW , Mullen GP , McManus JR , Heatherly JM , Duke A , Rand JB . Neuroligin‐deficient mutants of *C. elegans* have sensory processing deficits and are hypersensitive to oxidative stress and mercury toxicity. Dis Models & Mech. 2010;3(5‐6):366‐376. 10.1242/dmm.003442 PMC4068633 20083577

[228] Tu H , Fan C , Chen X , et al. Effects of cadmium, manganese, and lead on locomotor activity and neurexin 2a expression in zebrafish. Environ Toxicol Chem. 2017;36(8):2147‐2154. 10.1002/etc.3748 28120348

[229] Luo J , Qiu Z , Chen J , et al. Maternal and early life arsenite exposure impairs neurodevelopment and increases the expression of PSA‐NCAM in hippocampus of rat offspring. Toxicology. 2013;311(3):99‐106. 10.1016/j.tox.2013.06.007 23811142

[230] Dey PM , Gochfeld M , Reuhl KR . Developmental methylmercury administration alters cerebellar PSA‐NCAM expression and Golgi sialyltransferase activity. Brain Res. 1999;845(2):139‐151. 10.1016/s0006-8993(99)01887-9 10536193

[231] Marchand G , Fliniaux I , Titran P , et al. Cadmium induces physiological and behavioral changes associated with 180 kDa NCAM lower expression and higher polysialic acid, in the African clawed *Xenopus laevis* tadpoles. Ecotoxicol Environ Safety. 2024;273:116119. 10.1016/j.ecoenv.2024.116119 38382347

[232] Wilson DT , Polunas MA , Zhou R , Halladay AK , Lowndes HE , Reuhl KR . Methylmercury alters Eph and ephrin expression during neuronal differentiation of P19 embryonal carcinoma cells. Neurotoxicology. 2005;26(4):661‐674. 10.1016/j.neuro.2005.01.020 15990172

[233] Dey PM , Burger J , Gochfeld M , Reuhl KR . Developmental lead exposure disturbs expression of synaptic neural cell adhesion molecules in herring gull brains. Toxicology. 2000;146(2‐3):137‐147. 10.1016/s0300-483x(00)00171-2 10814846

[234] Burger J , Gochfeld M . Lead and neurobehavioral development in gulls: a model for understanding effects In the laboratory and the field. Neurotoxicology. 1997;18(2):495‐506. 9291497

[235] Bonfanti L , Theodosis DT . Polysialic acid and activity‐dependent synapse remodeling. Cell Adh Migr. 2009;3(1):43‐50. 10.4161/cam.3.1.7258 19372729 PMC2675148

[236] Senkov O , Tikhobrazova O , Dityatev A . PSA‐NCAM: synaptic functions mediated by its interactions with proteoglycans and glutamate receptors. Int J Biochem Cell Biol. 2012;44(4):591‐595. 10.1016/j.biocel.2012.01.008 22300986

[237] Wilson DT . Effects of Methylmercury on Ephs and ephrins During Central Nervous System Development. Rutgers The State University of New Jersey, School of Graduate Studies; 2003.

[238] Reuben KE , Parish A . Dissociation in Autism Spectrum Disorders: An Under‐Recognized Symptom. In: Christensen E, ed. Perspectives of Dissociativeidentity Response: Ethical, Historical, and Cultural Issues. HWC Press; 2022:151‐183.

[239] Murphy KJ , Regan CM . Low‐level lead exposure in the early postnatal period results in persisting neuroplastic deficits associated with memory consolidation. J Neurochem. 1999;72(5):2099‐2104. 10.1046/j.1471-4159.1999.0722099.x 10217290

[240] Heidmets L , Zharkovsky T , Jurgenson M , Jaako‐Movits K , Zharkovsky A . Early post‐natal, low‐level lead exposure increases the number of PSA‐NCAM expressing cells in the dentate gyrus of adult rat hippocampus. Neurotoxicology. 2006;27(1):39‐43. 10.1016/j.neuro.2005.05.015 16169083

